# DUSP8 as a regulator of glioblastoma stem-like cell contribution to tumor vascularization

**DOI:** 10.1186/s13046-025-03515-3

**Published:** 2025-09-30

**Authors:** Giorgia Castellani, Mariachiara Buccarelli, Quintino Giorgio D’Alessandris, Gabriele De Luca, Ramona Ilari, Francesca Pedini, Maurizio Martini, Cristiana Mollinari, Claudio Tabolacci, Gabriele Ricciardi, Emanuela Germanà, Valentina Lulli, Alessandra Boe, Mauro Biffoni, Giovanna Marziali, Roberto Pallini, Lucia Ricci-Vitiani

**Affiliations:** 1 Department of Oncology and Molecular Medicine, Istituto Superiore Di Sanità, Viale Regina Elena 299, 00161 Rome, Italy; 2 Department of Neurosurgery, Fondazione Policlinico Universitario A. Gemelli IRCCS, Rome, Italy; 3 Institutes of Neuroscience, Neurosurgery Section, Catholic University School of Medicine, Rome, Italy; 4 Department of Human Pathology of Adult and Developmental Age, “Gaetano Barresi”, Division of Pathology, Messina, Italy; 5 Department of Neuroscience, Istituto Superiore Di Sanita’, Rome, Italy; 6 Institute of Translational Pharmacology, National Research Council, Rome, Italy; 7 Research Coordination and Support Service, Istituto Superiore Di Sanità, Rome, Italy; 8 Department of Biomedical, Dental, Morphological and Functional Imaging Sciences, University of Messina, Messina, Italy; 9 Istituto Clinico Polispecialistico C.O.T Cure Ortopediche Traumatologiche S.P.a, Messina, Italy; 10 Core Facilities, Istituto Superiore Di Sanità, Rome, Italy

**Keywords:** Glioblastoma, Glioblastoma Stem-like cells, Endothelial transdifferentiation, MiR-1825, DUSP8, MAPKs

## Abstract

Glioblastomas (GBMs) are highly vascularized cancers. Transdifferentiation of GBM stem-like cells (GSCs) into GSC-derived endothelial cells (GdECs) contributes to GBM neovascularization. To dissect the molecular mechanisms and the signaling pathways underlying GSC contribution to tumor vascularization, we identified a three miRNA signature able to discriminate GSCs from GdECs by regulating different signaling pathways. DUSP8 resulted as the common target of the miRNA signature identified and is negatively regulated by miR-1825. DUSP8 is emerging as a critical negative regulator MAPKs pathway and is involved in cell oxidative stress response and apoptosis, as well as, in several diseases, including cancer. In GBM patients, DUSP8 and miR-1825 expression are inversely correlated and DUSP8 down-regulation is significantly associated with higher microvascular density and poor overall survival. Exploring the impact of DUSP8 in GSC transdifferentiation, we demonstrated that DUSP8 down-regulation interferes with MAPK pathway and affects soluble factor release. In vitro DUSP8 modulation experiments showed that DUSP8 enforced expression impairs GdEC ability to form tube-like structures. Gene expression variations induced by DUSP8 modulation affect transcripts associated with EMT pathway, confirming that DUSP8 shutdown and, therefore, the activation of MAPK pathway, is mandatory to GSC transdifferentiation. In vivo experiments demonstrated that both DUSP8 enforced expression and silencing dramatically affect gliomagenesis. Dissecting the molecular mechanisms underlying the contribution of GSCs to tumor angiogenesis might represent a chance to develop new and more efficient antiangiogenic therapeutic protocols for GBM treatment. Our findings provide a strong rationale to develop therapeutic strategies based on modulation of DUSP8 for GBM treatment.

**Supplementary Information:**

The online version contains supplementary material available at 10.1186/s13046-025-03515-3.

## Introduction

Glioblastoma, IDH-wildtype (GBM) is the most aggressive primary malignant brain tumor in adults. Despite the aggressive therapeutic strategies including maximal resection followed by chemoradiotherapy and, in some cases, antiangiogenic agents [1–3], the outcome of GBM patients remains poor [4]. Aberrant neoangiogenesis is a prominent feature of primary GBM, where the hypoxia represents the major driving force of tumor angiogenesis, determining a massive up-regulation of several pro-angiogenic factors, including the vascular endothelial growth factor (VEGF) [5, 6]. Antiangiogenic therapies targeting VEGF have therefore generated considerable interest over the years. In 2009, the Food and Drug Administration (FDA) approved the humanized anti-VEGF monoclonal antibody, bevacizumab, as a single agent to treat recurrent GBM. However, phase III clinical trials both in newly diagnosed GBM and in recurrent GBM [7] demonstrated that bevacizumab does not prolong patients’ overall survival (OS), despite the extension of progression-free survival (PFS). It is emerging that neovascularization in GBM is a complex, dynamic, and heterogeneous process, where the VEGF-mediated angiogenesis coexists with numerous non-canonical strategies, variously exploited by GBM to meet its ever-increasing metabolic demand, and differently involved in tumor progression, recurrence, and escape from treatments [8]. Moreover, recent studies have suggested that the microvasculature of GBM may vary depending on tumor type, tumor cell heterogeneity, and tumor stage [9].

In this way, we have demonstrated that glioblastoma stem-like cells (GSCs), a subpopulation of tumor initiating stem-like cells capable of supporting malignant properties including initiation, growth, resistance to therapies and tumor recurrence [10], are able to differentiate in functional endothelial-like cells, contributing to the tumor vasculature [11]. Moreover, we demonstrated that selective targeting of GSC-derived endothelial cells (GdECs) in mouse xenografts resulted in tumor reduction and degeneration, highlighting the functional relevance of the GSC-derived endothelium and confirming the existence of a new mechanism of tumor angiogenesis [11]. The contribution of GSCs to vasculogenesis by transdifferentiating into endothelial-like cells, may provide a mechanism for evading VEGF inhibition.

Through their ability to regulate many genes, microRNAs (miRNAs), a class of short non-coding RNAs, has been shown to control diverse oncogenic signaling pathways including GSC behavior, cell differentiation and angiogenesis [12].

In the present study we identified a three miRNA signature that clearly distinguish GSCs cultivated in stem cell medium or differentiated in endothelial conditions and are able to regulate GSC-contribution to tumor vascularization through dual-specificity phosphatase 8 (DUSP8) modulation. DUSP8 is emerging as a critical negative regulator for Mitogen-activated protein kinase (MAPK) pathway and it is involved in cell oxidative stress response and cell apoptosis, as well as the development of various human diseases, including cancer [13].

## Methods

### Cell cultures

GSCs were isolated from surgical samples of adult patients who underwent craniotomy at the Department of Neurosurgery, Fondazione Policlinico Universitario A. Gemelli IRCCS, Rome, upon approval by the local ethical committee (Prot. ID 2253 and 3782). Informed consent was obtained from the patients before surgery. Clinical features of GBM patients and tumors are shown in Supplementary Table S1. Information of GSC lines, HeLa and 293 T cells are described in the Supplementary Methods.

### Transdifferentiation of GSCs

GSCs were cultured in endothelial conditions, as previously described [14]. The detailed methods are described in Supplementary Methods.

### Flow cytometry

Evaluation of the expression of endothelial markers was performed as previously described [14]. The detailed methods are described in Supplementary Methods.

### RT-PCR

Total RNA was isolated from cells using TRIzol reagent (Invitrogen by ThermoFisher Scientific). RNA was reverse transcribed by TaqMan MicroRNA Reverse Transcription Kit (Applied Biosystems™ by ThermoFisher Scientific, Waltham, MA, USA) and LunaScript® RT SuperMix Kit (New England Biolabs, Ipswich, MA, USA).

Detailed information on RT-PCR for miRNAs and DUSP8 mRNA detection are available inSupplementary Methods.

### Automated capillary Western immunoassay (WES)

Total protein lysates for WES were performed as previously described [15]. See Supplementary Methods for the list of primary antibodies used.

### Luciferase Reporter Assay

Detailed information on luciferase reporter assay are described in Supplementary Methods.

### RNA Immunoprecipitation (RIP)

RIP assay was performed by using the miRNA Target IP Kit (Active Motif, Carlsbad, CA, USA) according to manufacturer’s instruction. Detailed methods are available in Supplementary Methods.

### Drug cytotoxicity experiments

For cytotoxicity experiments, GSCs were mechanically dissociated and plated in a 96-well plate, in triplicate, at a density of 2 × 10^4^ cells/ml. GdECs were plated at a density of 1 × 10^4^ cells/ml in a 96-well plate. Ulixertinib, trametinib and ralimetinib were purchased from SelleckChem (Selleck chemicals, Houston, TX, USA). Compounds were dissolved in DMSO and added 24 h after cell plating at indicated concentrations ranging from 1.25 µM to 5 µM for ulixertinib, 75 nM to 300 nM for trametinib, and 10 µM to 40 µM for ralimetinib. ATP levels were measured using the CellTiter-Glo™ (Promega Inc., Madison, WI, USA) according to the manufacturer’s instructions, as previously reported [14].

### Plasmid constructs and lentivirus infection

For DUSP8 silencing and ectopic expression experiments, short hairpin (sh)-DUSP8-Green Fluorescent Protein (GFP) (TL313344) and DUSP8-GFP (RC208337) lentiviral vectors were purchased from OriGene Technologies (Rockville, MD, USA).

Lentiviral particles were produced in 293 T at 70–80% confluency, using the calcium phosphate transfection protocol and infection was performed as previously reported [16].

### Analysis of conditioned medium by Luminex assay

GSCs and GdECs were cultured as previously described. After 48 h of incubation under standard condition, cells were counted and conditioned media were collected and centrifuged to remove cell debris. Conditioned media were analyzed with a magnetic bead-based multiplex immunoassay on the Luminex platform as previously described [17]. The detailed methods and the list of selected soluble molecules are described in Supplementary Methods.

### Microarrays and RNA-Sequencing analysis (RNA-Seq)

To analyze miRNA expression, total RNA was prepared using Qiagen RNeasy Micro Kit (Qiagen, Hilden, Germany). 1 µg of RNA was labeled and hybridized to the Agilent-019118 array (Agilent Technologies, Santa Clara, CA, USA) following the manufacturer's instructions.

RNA-Seq on transduced GSCs and GdECs was performed by Genomix4life (Laboratory of Molecular Medicine and Genomics, University of Salerno). See Supplementary Methods for detailed information.

### Tube formation assay

Detailed information on tube formation assay are described in Supplementary Methods.

### Intracranial implantation of GSCs into immunocompromised mice

Animal experiments were performed in accordance to relevant institutional and national regulations (Aut.n. 53/2023-PR, prot. D9997.150).

NOD-SCID mice (*n* = 12 males; 4–6 week old; Charles River, Italy) were implanted intracranially with 2 × 10^5^ GFP, DUSP8-GFP and sh-DUSP8-GFP expressing GSC#1 line (see Supplementary Methods).

### Immunofluorescence analysis of tumors in brain slices

For information on immunofluorescence analysis see Supplementary Methods.

### Immunohistochemistry (IHC), microvessel density evaluation (MVD) and Combined in situ Hybridization/Immunohistochemistry (ISH/IHC)

Detailed information on IHC, MVD and combined ISH/IHC are available in Supplementary Methods.

### GBM patient cohort

For more information about the cohort included in the study see Supplementary Methods.

### Statistical analysis

Detailed information of all the statistical analyses performed is described in Supplementary Methods.

## Results

### Three miRNA signature discriminates GSCs from GdECs by regulating different signaling pathways

GSCs are directly involved in new vessel formation via their transdifferentiation into tumor-derived endothelial cells. As previously showed [14], GSCs cultured under hypoxia in endothelial conditions, i.e. stem cell medium with serum and endothelial growth factors, unlike GSCs grown under normoxia in stem cell medium, generate tube-forming endothelial-like cells, which show a consistent increase of CD34, widely regarded as a marker of vascular endothelial progenitor cells [18].

**Fig. 1:**
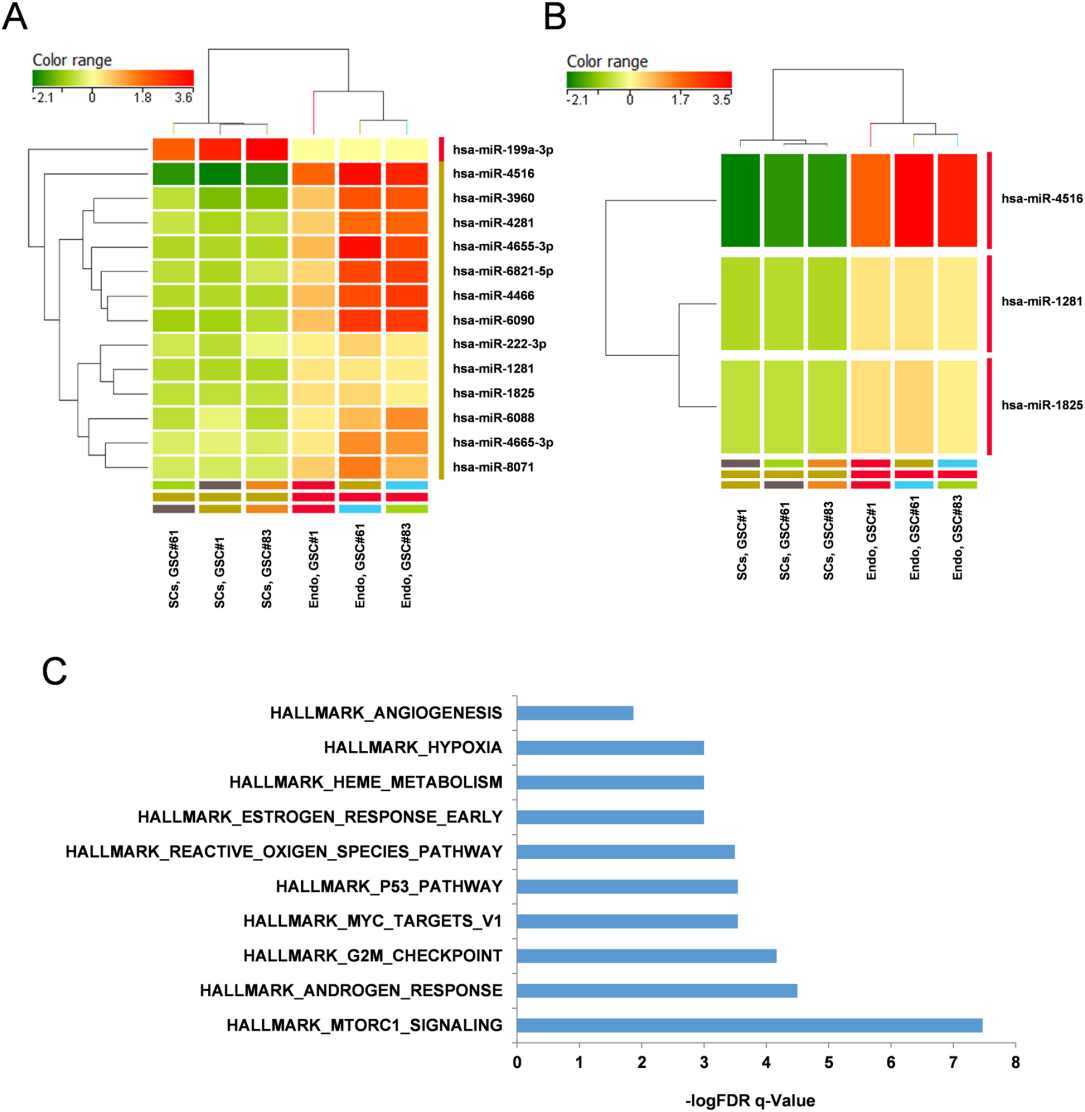
miRNA expression pattern analysis in patient-derived GSCs and GdECs. **A** Heatmap shows the results of unpaired hierarchical clustering of global miRNA expressions. This analysis identifies a signature of fourteen miRNAs, modulated in GdECs compared to GSCs and capable of distinguishing between the two clusters. Color scale represents miRNA expression levels. **B** Heatmap shows the results of paired hierarchical clustering of global miRNA expressions. This analysis identifies a signature of three miRNAs (i.e. miR-1281, miR-1825, miR-4516), up-regulated in GdECs compared to GSCs and capable of distinguishing between the two clusters. Color scale represents miRNA expression levels. **C** GSEA of the miRNA targets. Genes modulated by the three miRNAs are associated with pathways related to angiogenesis, hypoxia and ROS metabolism

In order to identify signaling pathways with potentially relevant functions in GdEC survival, we performed miRNA profiling of the three GSC lines, namely GSC#1, GSC#61 and GSC#83, either cultivated in stem cell medium (SCs) or in endothelial conditions (Endo). Hierarchical clustering of global miRNA expression pattern revealed two distinct clusters: the so-called"SCs"and the"Endo"clusters. The signature underlying SCs *vs* Endo clustering included a list of 14 miRNAs differentially expressed between the two clusters (Fig. 1A). Then, paired hierarchical clustering revealed a signature of three miRNAs, miR-4516, miR-1281 and miR-1825, up-regulated in GdECs compared to GSCs and able to clearly distinguish GSCs cultivated in stem cell medium or differentiated in endothelial conditions (Fig. 1B).

Gene Set Enrichment Analysis (GSEA) of the three miRNA targets revealed a modulation of genes associated with pathways involved in different processes such as angiogenesis, hypoxia, reactive oxygen species (ROS) metabolism (Fig. 1C). These results suggest a possible implication of these three miRNAs into the GSC-associated neovascularization process.

### DUSP8 is the common target of the three miRNAs but is negatively regulated by miR-1825 in GSCs

**Fig. 2:**
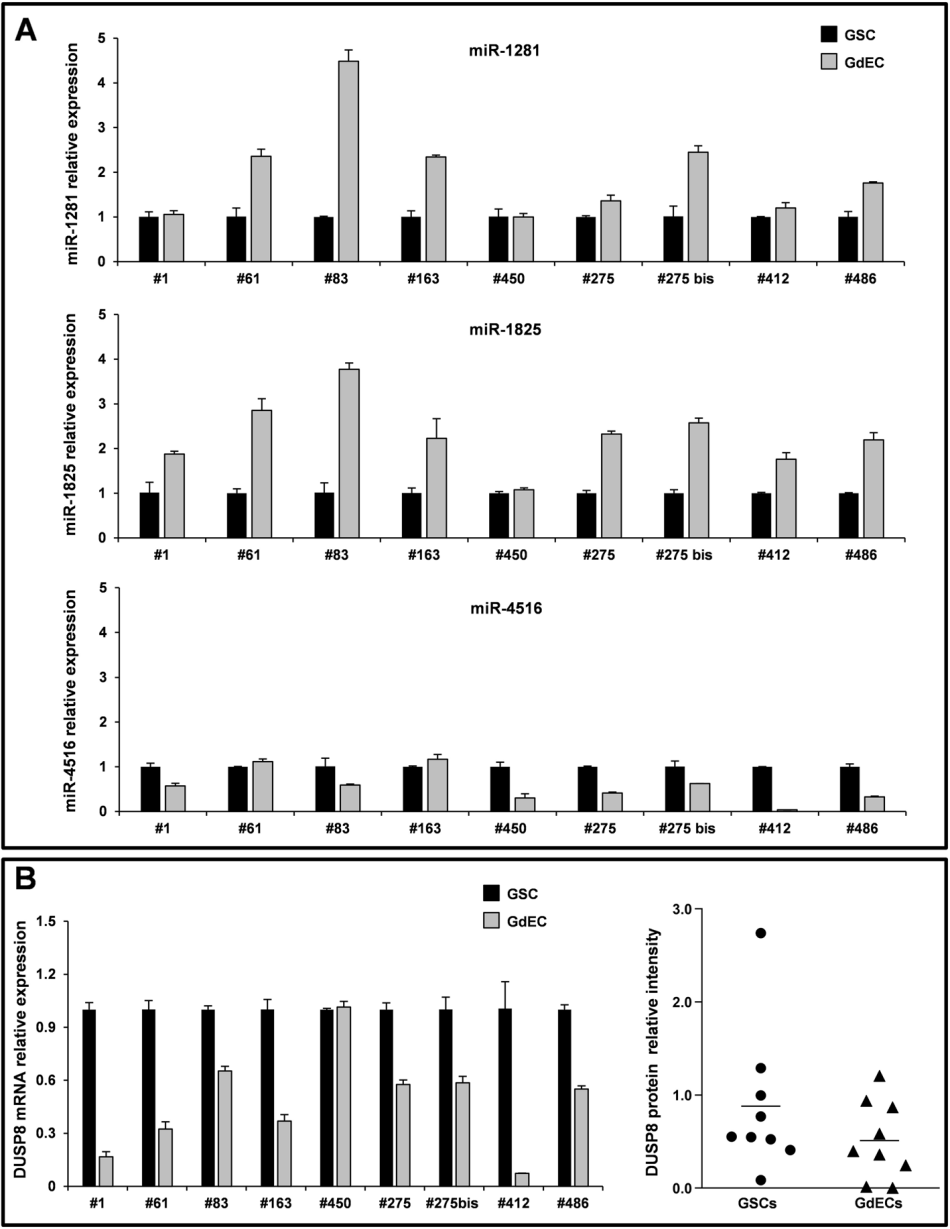
Validation of the up-regulation of the three miRNAs and of the down-regulation of DUSP8 in GdECs compared to GSCs. **A** RT-PCR analysis of the three miRNAs (i.e. miR-1281, miR-1825, miR-4516), confirms the up-regulation of miR-1281 and miR-1825 in GdECs compared to GSCs (*n* = 9). Samples were run in duplicate. Data were normalized to the small nuclear RNA RNU6B expression in the corresponding samples. **B** RT-PCR and WES analysis show the down-regulation of DUSP8 mRNA (*left panel*) and protein (*right panel*) in GdECs compared to GSCs (*n* = 9). For RT-PCR analysis, samples were run in duplicate. Data were normalized to the GAPDH expression in the corresponding samples. DUSP8 protein values were quantified using AUC measurements. Signal intensity was normalized to the µ-actin expression in the same samples

To confirm the implication of the three miRNAs in endothelial transdifferentiation of GSCs, we compared the expression of miR-1281, miR-1825 and miR-4516 in GSC lines cultured under normoxia, in stem cell medium, or under hypoxia, in endothelial conditions, two weeks after seeding. Particularly, we analyzed by RT-PCR, the three GSC lines used for the miRNA profiling (i.e. GSC#1, GSC#61 and GSC#83) and a larger panel of patient-derived GSCs, including two pairs of GSC lines derived from the same patient at primary (GSC#275, GSC#412) and secondary surgery (GSC#275 bis and GSC#486), confirming the up-regulation of miR-1281 and miR-1825 in endothelial condition (Fig. 2A). Conversely, miR-4516 expression decreased after endothelial differentiation in most of the cell lines tested, including two out of the three GSC lines used for the miRNA profiling.

A list of the potential target mRNAs for miR-4516, miR-1281 and miR-1825 was obtained using miRBase (http://mirbase.org/) algorithm [19]. Among these, the dual-specificity phosphatase 8 (DUSP8), belonging to the phosphatases family, known for their ability to dephosphorylate both tyrosine and serine/threonine residues [20], is the only common target of the three miRNAs. The comparison between GSCs and corresponding GdECs for the expression of the potential target of the three miRNAs, DUSP8, revealed that its expression decreased after endothelial transdifferentiation, except for GSC#450 (Fig. 2B, *left*). Accordingly, cytofluorimetric evaluation of endothelial markers (i.e. CD31, CD34 Tie2, VEGFR2/KDR and CD133) in GSC#450 two weeks after culture in endothelial conditions, revealed that differently from the other GSC lines analyzed, endothelial marker expression did not increase, except for CD31, suggesting an incomplete endothelial transdifferentiation (Supplementary Fig. S1).

In line with RT-PCR analysis results, DUSP8 protein levels decreased after endothelial transdifferentiation as well (Fig. 2B, *right*).

Dual-luciferase reporter assays were performed to prove the direct binding between DUSP8 and miR-4516, miR-1281 and miR-1825 (Supplementary Fig.S2).

The human DUSP8 3′-UTR contains four target sites for miR-1281, two for miR-1825 and three for miR-4516 (Supplementary Fig. S2A). To better verify the direct binding, we divided DUSP8 3′-UTR into two regions. Depending on the region we performed luciferase assays by transfecting 293 T cells with single hsa-miR-1281 or hsa-miR-4516 or both hsa-miRs (Supplementary Fig. S2B, *left*), and hsa-miR-1281 or hsa-miR-1825 or hsa-miR-4516 or all the three hsa-miRs (Supplementary Fig. S2B, *right*). Among the three miRNAs that may potentially regulate DUSP8 expression, the luciferase assay showed that only miR-1825 is able to significantly bind to DUSP8 3’-UTR (at position 1790) reducing by about 50% luciferase level (Supplementary Fig. S2C).

To confirm the putative direct binding between miR-1825 and DUSP8 we performed RIP using a pan-Ago antibody to pulldown the endogenous DUSP8 associated to miRNA by using HeLa cells transiently transfected with hsa-miR-1825 mimic or scramble (Supplementary Fig. S2D). RT-PCR analysis on RNA recovered after RIP assay showed a fold-enrichment for DUSP8 between the hsa-miR-1825 mimic or scramble-transfected HeLa cells, confirming the ability of miR-1825 to negatively modulate DUSP8 expression by direct binding of DUSP8 mRNA.

*DUSP8 and miR-1825 expression are inversely correlated and DUSP8 down-regulation is significantly associated with higher microvascular density and poor overall survival of GBM patients.*

**Fig. 3:**
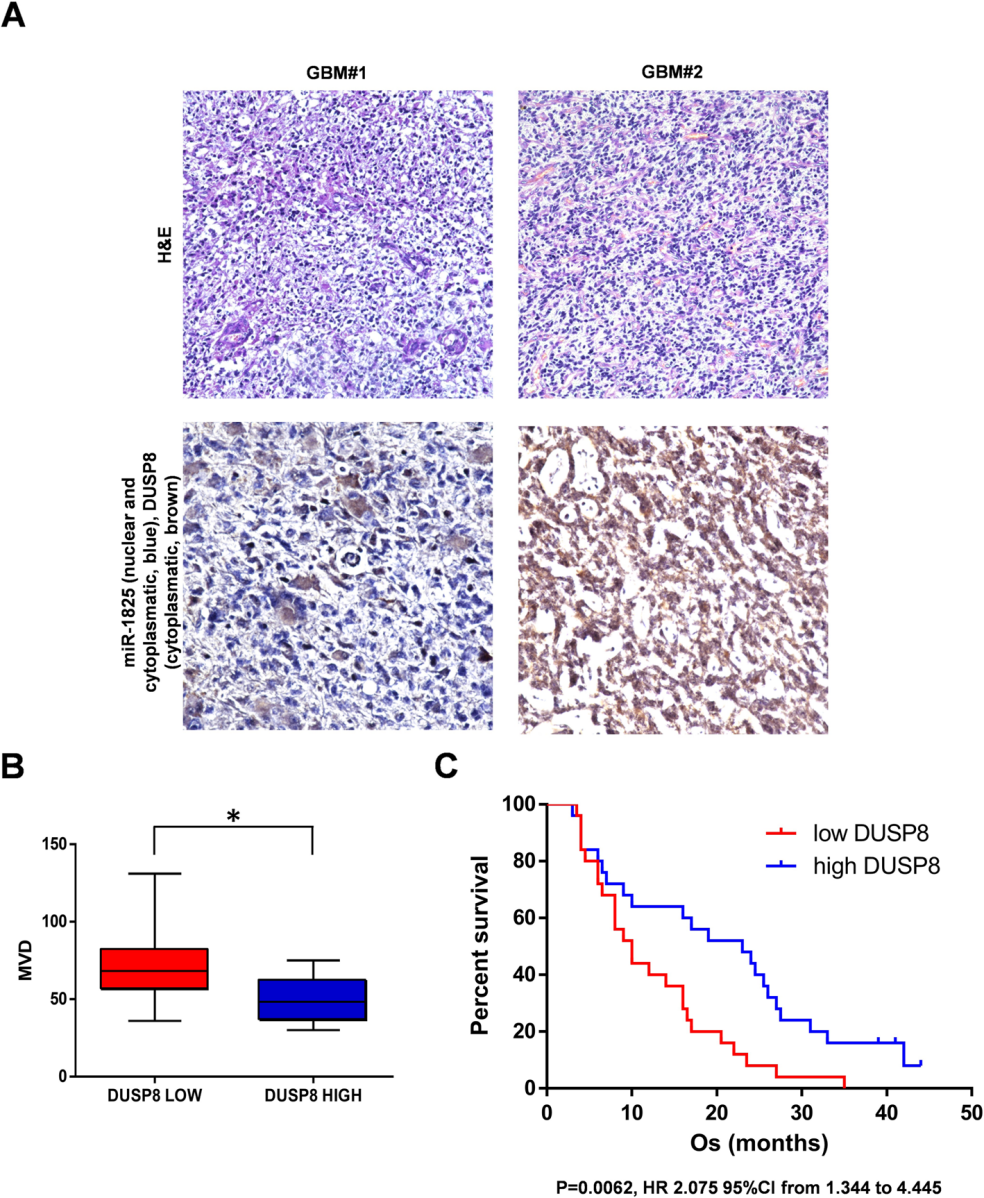
Study of miR-1825 and DUSP8 expression in GBM patients. **A** Combined in situ hybridization/immunohistochemistry (ISH/IHC) of two exemplificative cases of GBM (H&E for GBM#1 and GBM#2 in the upper pictures, 100X magnification) showing the inverse correlation between miR-1825 and DUSP8 in the glial cell tumor (lower pictures, 200X magnification). **B** Whiskers box plots show significant higher MVD in GBMs with lower DUSP8 expression in comparison to the GBMs with higher DUSP8 expression (*p* = 0.041; Mann–Whitney U test). **C** Kaplan–Meier survival curves of 50 GBM patients stratified by DUSP8 expression. The high level of DUSP8 expression in tumors (*blue line*) conferred a favorable survival advantage in comparison with those GBMs with low DUSP8 expression (*red line*) (*p* = 0.0062, HR 2.075 95%CI from 1.344 to 4.445)

To verify the correlation between DUSP8 and miR-1825 expression in GBM patients, we performed a combined in situ hybridization/immunohistochemistry (ISH/IHC) for miR-1825/DUSP8 on 8 GBM cases (4 with high and 4 with low expression of DUSP8). All cases analyzed showed an inverse relation between DUSP8 and miR-1825 (Fig. 3A). Specifically, glial tumor cells with high expression of DUSP8 (cytoplasmic expression) showed low expression of miR-1825 (cytoplasmic and nuclear expression), while glial cells with low DUSP8 expression showed higher expression of miR-1825 (Fig. 3A, *lower panel*).

Microvascular Density (MVD) analysis of GBMs (*n* = 24) showed that microvessel counts (using CD31 staining) in GBMs with lower DUSP8 expression were significantly higher respect to GBMs with higher DUSP8 expression (71.9 ± 11.0 *vs* 49.1 ± 8.2, mean ± SD; *p* = 0.041, Mann–Whitney U test) (Fig. 3B).

The mean OS of our cohort of IDH-wild type GBM patients (*n* = 50) was 16.5 months (range 3–42 months). OS was not influenced by age, sex, surgical resection (partial *vs* total), Ki67, and p53 (*p* = 0.06, *p* = 0.64. *p* = 0.72, *p* = 0.42 and *p* = 0.99, respectively; Kaplan–Meier survival analysis, Logrank test, Supplementary Fig. S3A). As expected, MGMT (*p* = 0.049, HR 0.54, 95% CI from 0.3 to 0.99; Supplementary Fig. S3B), and KPS (*p* = 0.0042, HR 0.41, 95% CI from 0.22 to 0.75; Supplementary Fig. S3C) turned out to be predictors of OS at univariable analysis (Kaplan–Meier survival analysis, Logrank test).

Interestingly, Kaplan–Meier analysis of our cohort of GBM patients indicated that lower expression of DUSP8 significantly correlated with shorter OS (*p* = 0.0062, HR 2.075 95%CI from 1.344 to 4.445) (Fig. 3C). Moreover, in the multivariate analysis, DUSP8 expression (*p* = 0.0242) and KPS (*p* = 0.0157) were independent predictors of OS (Supplementary Fig. S3D; Cox proportional-hazards regression analysis).

### Endothelial transdifferentiation-induced DUSP8 down-regulation interferes with MAPK pathway and affects soluble factor secretion

**Fig. 4:**
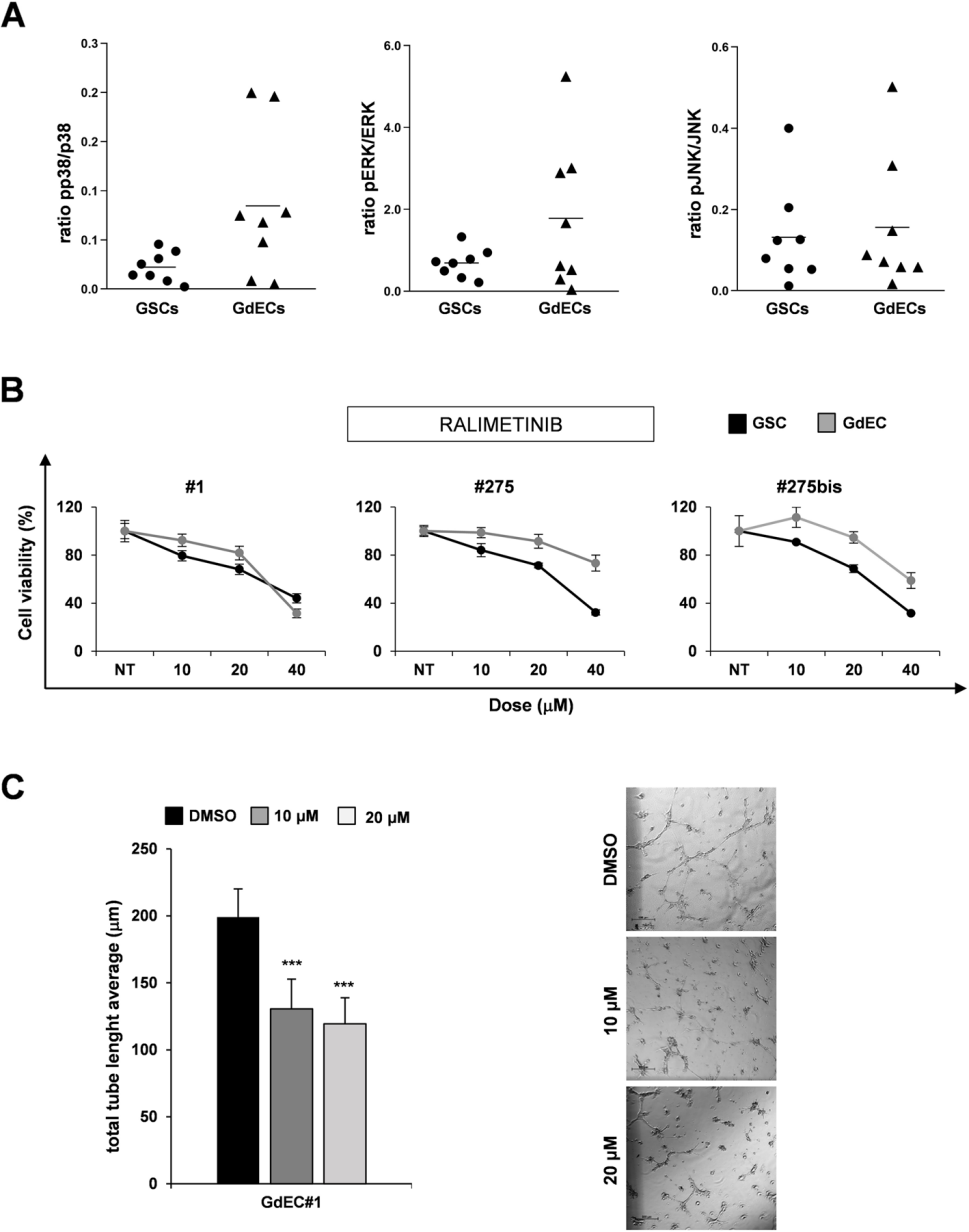
DUSP8 modulation interferes with MAPK pathway during transdifferentiation. **A** WES analysis shows the pp38/p38 ratio, pERK/ERK ratio and pJNK/JNK ratio in a panel of 8 GdEC lines and their counterpart GSCs. The protein expression values were quantified using AUC measurements. Signal intensity was normalized to the β-actin expression in the same sample. **B** Concentration-response assays of ralimetinib were performed on three representative patient-derived cell lines (i.e. #1, #275, #275bis) either in stem cell medium (GSC) or in endothelial conditions (GdEC). For each dose, mean values ± SD of residual cell viability normalized to non-treated (NT) cells are reported and drug tested for 48 h. **C** Tubule formation assay performed on GdEC#1 treated with vehicle or different doses of ralimetinib (10 µM and 20 µM); quantification of total tube length average is reported (*left*). Representative pictures of tube-like structures in vehicle-treated GdEC#1 (DMSO), and GdEC#1 treated with ralimetinib at 10 µM and 20 µM formed after 6 h (*right*). Scale bar: 500 μm. Total magnification 2X. ***p < 0.001 (Student’s *t* test)

The potential role of DUSP8 in GSC endothelial transdifferentiation could be mainly exerted by its dephosphorylation activity on phosphorylated serine, threonine, and tyrosine residues of the ERK, and, particularly, p38 and JNK substrates [21, 22]. Therefore, we investigated the impact of DUSP8 decrease occurring during the GSC transdifferentiation process on MAPK pathway. We evaluated the phosphorylation status of p38, ERK 1/2 and JNK, as substrates of DUSP8 activity, in eight GSC lines and their GdEC counterpart. We found an increase in kinase phosphorylation levels of both p38, ERK 1/2 and JNK in GdECs compared to GSCs, suggesting the activation of MAPK pathway during GSC endothelial transdifferentiation (Fig. 4A).

To verify whether interfering with MAPK pathway activation could impair GSC endothelial transdifferentiation, we tested in vitro the effects of a set of MAPK pathway inhibitors, already approved for clinical trials or for therapy, on GSC#1, matched primary and secondary surgery GSC#275 and GSC#275bis lines, and their GdEC counterpart, showing different phosphorylation status of MAPK pathway (Supplementary Fig. S4A). Particularly, we had chosen: ralimetinib (LY2228820), a selective inhibitor of α- and β-isoforms of p38-MAPK tested in phase I trial with radiotherapy plus concomitant TMZ in the treatment of newly diagnosed GBM [23]; ulixertinib (BVD-523), a reversible, ATP-competitive, catalytic ERK1/2 inhibitor, currently tested in a clinical phase II trial with pediatric patients with MAPK pathway mutations and pediatric patients with relapsed solid and brain tumors [24]. Unfortunately, a selectively inhibitor of JNK approved for clinical trials has not been found; so we chose trametinib, an inhibitor of MEK1/2, an upstream effector of p38, JNK and ERK1/2, that is being investigated in the treatment of pediatric low-grade gliomas and plexiform neurofibroma in an ongoing multicenter phase II trial [25] (Fig. 4B and Supplementary Fig. S4B). As expected, GdECs showed lower sensitivity to these compounds compared to GSCs. Moreover, only ralimetinib was able to impair GdEC viability in all the three cell lines tested (Fig. 4B and Supplementary Fig. S4B). The result of cytotoxicity experiments was corroborated by the tubule formation assay performed on GdEC#1 treated with ralimetinib at different doses (10 μM and 20 μM). After 6 h, treated cells showed a significantly lower ability to form tube-like structures compared to vehicle-treated cells (DMSO) (Fig. 4C), whereas ulixertinib and trametinib treatment did not affect tube-like structures formation (Supplementary Fig. S4C).

To deepen the lack of response to ulixertinib and trametinib, we evaluated pERK/ERK 48 h after ulixertinib treatment and we observed an increase of pERK/ERK ratio in both GSC#1 and GdEC#1 (Supplementary Fig. S5, *left panel*). This observation, in itself, does not justified the drug's ineffectiveness in inhibiting GdEC#1 cell growth, because it has been described in acute leukemia cell lines that ulixertinib inhibits ERK-mediated signal transduction without altering protein expression or phosphorylation [26]. Accordingly, ERK pathway blocking was confirmed in GSCs by growth inhibition whereas, in GdECs, where we observed more than twofold increase in basal pERK/ERK ratio compared to GSCs (Supplementary Fig. S4A), ulixertinib inhibition determined a furher accumulation of pERK without inhibition of growth, suggesting a reduced addiction of GdECs to downstream ERK pathway compared to GSC counterpart.

Treatment for 48 h with trametinib efficiently inhibited ERK phosphorylation but, once again, this inhibition was able to interfere with GSC but not with GdEC growth confirming their less addiction to MEK/ERK pathway (Supplementary Fig. S5, *right panel*).

These results suggest that interfering with MAPK pathway through the p38 inhibitor ralimetinib impairs GSC transdifferentiation ability, underlying the role of MAPK pathway in this process.

Moreover, it has been recently demonstrated ralimetinib ability to inhibit EGFR kinase activity in vitro acting as an ATP-competitive EGFR inhibitor [27]. It has been supposed that even though ralimetinib is > 30-fold less potent against EGFR compared to p38, it has off-target activity against EGFR, which could suggest that the effects observed in our system are not limited to p38 inhibition.

The relationship between GSCs and surrounding microenvironment is crucial for regulating self-renewal and angiogenesis in GBM [28]. We then measured a panel of soluble secreted molecules in GSC and GdEC supernatants, including cytokines/chemokines, growth factors, and matrix metalloproteinases (MMPs) among others. We found that most of the analyzed molecules are up-regulated in GdECs with respect to GSC counterpart (Supplementary Fig. S6A). Among these, the key secretory molecules, IFN-γ, CXCL10, IL-4, VEGF, and TNF-α, are mainly involved in angiogenesis and tumor progression (Supplementary Fig. S6B) [29–31]. The observed up-regulation of all MMPs might corroborate the pro-tumorigenic aptitude of GdECs. Moreover, we observed also an increasing level of ICAM-1 and VCAM-1, strongly related to glioma clinico-pathological grade [32].

### Modulation of DUSP8 expression affects endothelial transdifferentiation ability of GSCs

To deeply investigate the role of DUSP8 in GSC transdifferentiation, we performed gene modulation experiments and evaluated the effects of DUSP8 ectopic expression or silencing on GSC transdifferentiation ability. To this end, we transduced GSC#1 by using a lentiviral vector carrying DUSP8 and GFP as reporter gene to obtain DUSP8 ectopic expression, or by using the most efficient sh-DUSP8-GFP lentiviral construct, out of four tested, to silence DUSP8 expression (Supplementary Fig. S7A-B).

**Fig. 5:**
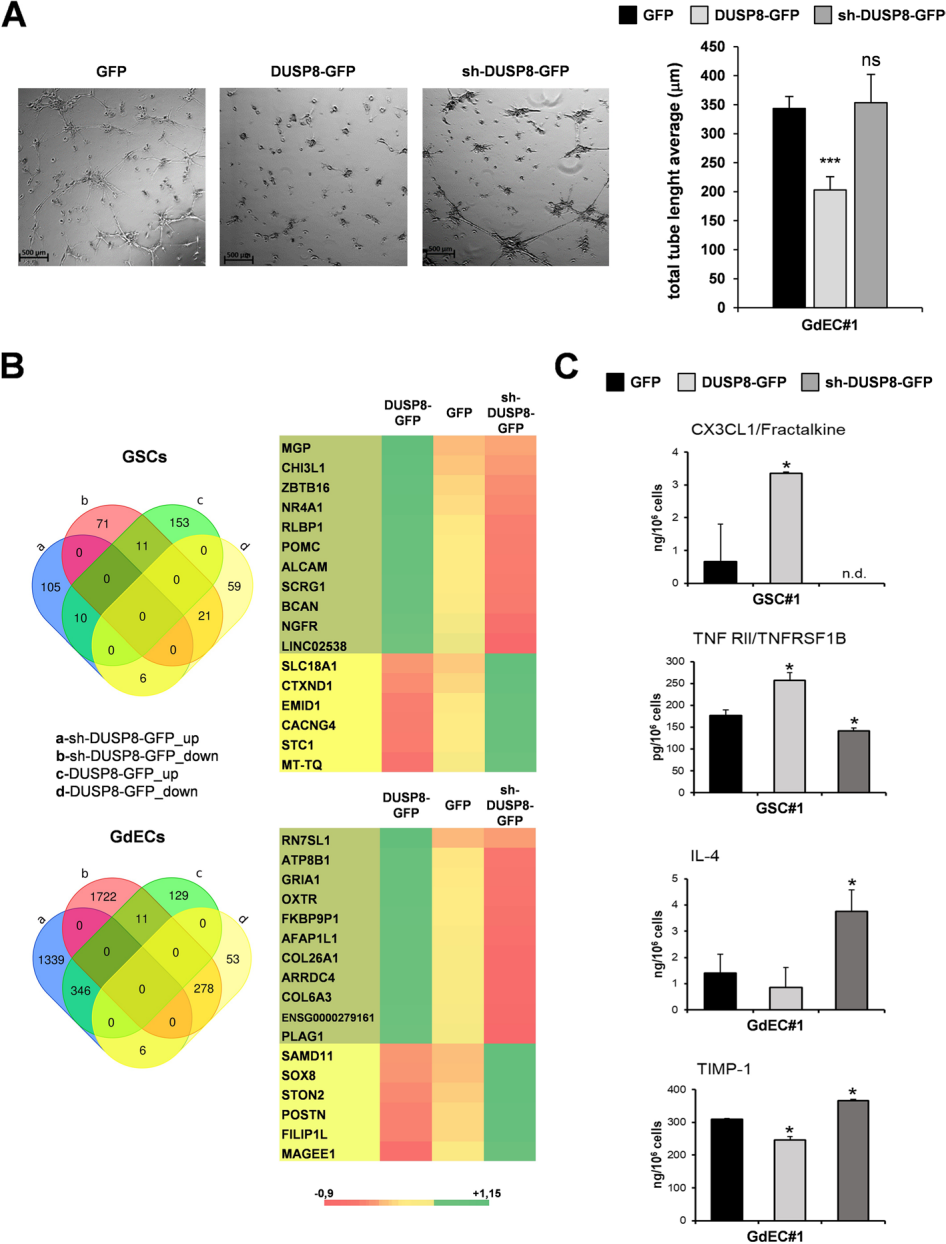
DUSP8 modulation affects endothelial transdifferentiation ability of GSCs. **A** Tubule formation assay performed on GdEC#1 transduced with GFP, DUSP8-GFP and sh-DUSP8-GFP. Representative pictures of tube-like structures in GdEC#1 transduced with GFP, DUSP8-GFP and sh-DUSP8-GFP formed after 6 h (*left*). The quantification of total tube length average is reported (*right*). Scale bar: 500 μm. Total magnification 2X. ns non-significant, ***p < 0.001 (Student’s *t* test). **B** Venn diagram constructed using differentially expressed genes from RNA-Seq data analysis in the GFP, DUSP8-GFP and sh-DUSP8-GFP transduced GSC#1 and GdEC#1 (*left*). Heatmap showing the most differentially expressed genes between GFP, DUSP8-GFP and sh-DUSP8-GFP transduced GSC#1 (*up*) and GdEC#1 (*bottom*). **C** Analysis of soluble molecules secreted by GSC#1 and GdEC#1 transduced with GFP, DUSP8-GFP and sh-DUSP8-GFP. Concentrations of selected analytes were quantified by Luminex immunoassay. Results are shown as mean ± SD of triplicate samples. n.d. not determined; *p < 0.05 (Student’s t test)

Noteworthy, DUSP8 ectopic expression impaired the ability of GdECs to form tube-like structures whereas, silencing did not, supporting the hypothesis of its involvement in endothelial transdifferentiation and angiogenesis-related features (Fig. 5A). Moreover, after DUSP8 enforced expression GdEC#1 showed an increased sensitivity to ralimetinib treatment relative to their GSC counterpart, compared to GdEC#1 GFP, whereas GdEC#1 sh-DUSP8-GFP showed a decreased sensitivity (Supplementary Fig. S8A). Once again sensitivity to ulixertinib and trametinib did not show any modulation either after DUSP8 ectopic expression or silencing (Supplementary Fig. S8A). This result suggests that the effects of ralimetinib treatment are mediated by DUSP8 regulation of MAPK pathway, as further confirmed by the reduced p38 activation (Supplementary Fig. S8B).

RNA-Seq analysis on DUSP8-GFP, sh-DUSP8-GFP and their relative GFP-control GSC#1 and GdEC#1 transduced cells showed that the differentially expressed genes (fold change > 1.5 and < −1.5) from RNA-Seq data analysis in the transduced GSC#1, were 224 (sh-DUSP8-GFP *vs* GFP) and 260 genes (DUSP8-GFP *vs* GFP), while in transduced GdEC#1, the differentially expressed genes were 3702 (sh-DUSP8-GFP *vs* GFP) and 823 (DUSP8-GFP *vs* GFP). A Venn diagram showed that the common genes in the up-regulated groups in GSC#1 transduced with sh-DUSP8-GFP (*vs* GFP) and the down-regulated groups in DUSP8-GFP (*vs* GFP) were only 6, while common genes in the downregulated in sh-DUSP8-GFP (*vs* GFP) and the up-regulated groups in DUSP8 GFP (*vs* GFP) were 11 (Fig. 5B). Among the transcripts down-regulated in DUSP8-GFP GSC#1 and up-regulated in sh-DUSP8-GFP GSC#1 we found stanniocalcin-1 (STC1), a secreted glycoprotein associated with poor prognosis, glioma grade, and resistance to TMZ therapy [33]. The same analysis was performed on DUSP8-GFP, sh-DUSP8-GFP and their relative GFP-control GdEC#1 transduced cells. The Venn diagram resulting from this analysis shows 11 common genes between those up-regulated in DUSP8-GFP *vs* control GFP, and down-modulated after DUSP8 silencing *vs* control GFP; only 6 are the common genes among those down-regulated in DUSP8-GFP and up-modulated in sh-DUSP8-GFP. Interestingly, among the transcripts down-regulated in DUSP8-GFP GdEC#1 and up-regulated in sh-DUSP8-GFP GdEC#1 we found periostin (POSTN) a matricellular protein that has been associated with glioma progression, particularly with glioma angiogenesis [34].

Furthermore, Luminex analysis in supernatants of DUSP8-GFP, sh-DUSP8-GFP and their relative GFP-control GSC#1 and GdEC#1 transduced cells revealed that DUSP8 modulation exerts opposite effects in a group of four secreted molecules, all implicated in the malignant behaviour of GBM. Particularly, in the GSC context, DUSP8 modulation exerts opposite effects on CX3CL1 and TNF RII secreted molecules, whereas, in GdECs, DUSP8 modulation affected the secretion of TIMP-1 and IL-4 soluble molecule in an opposite way (Fig. 5C).

### Modulation of DUSP8 expression affects gliomagenesis in vivo

**Fig. 6:**
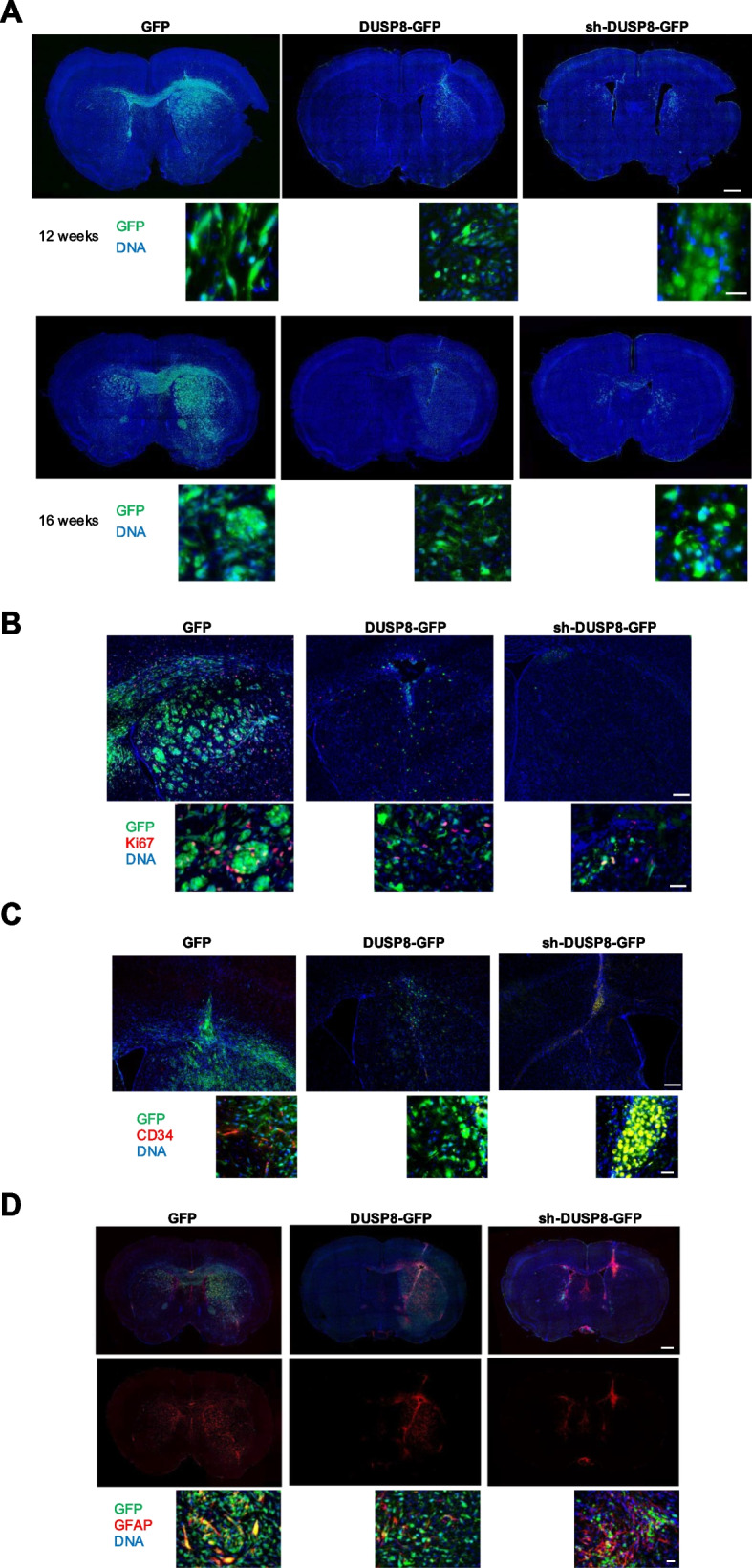
In vivo DUSP8 modulation inhibits tumor growth in NOD-SCID mouse brain. **A** GFP, DUSP8-GFP and sh-DUSP8-GFP GSC#1 cells were injected into the right brain hemisphere of NOD/SCID mice. Representative images of coronal mouse brain sections at 12 and 16 weeks (*upper and lower panel*, respectively) after the intracranial injection of GSCs. Free floating brain slices were counterstained with DAPI (*blue*). Scale bar: 500 μm. Insets show GFP^+^ cell morphology. Scale bar: 50 μm. **B** Representative triple immunofluorescence images of coronal brain slices stained using DAPI (*blue*) and an antibody specific for Ki67 (*red*). A reduction in the number of dividing cells is observed both in DUSP8-GFP and sh-DUSP8-GFP brain slices. Scale bar: 250 μm. Insets show a higher magnification of GFP^+^ cells. Scale bar: 50 μm. **C** Representative triple immunofluorescence images of coronal brain slices stained using DAPI (*blue*) and an antibody specific for CD34 (*red*). In control GFP brain slices, CD34^+^ GFP^+^ cells are highly dispersed within tumor mass in both hemispheres and show an elongated morphology. In DUSP8-GFP coronal slices CD34^+^ GFP^+^ cells scarcely detectable, whereas, in sh-DUSP8-GFP coronal slices, almost all GFP^+^ cells express CD34. Scale bar: 250 μm. Insets show a higher magnification of GFP^+^ cells. Scale bar: 50 μm. **D** Representative triple immunofluorescence images of coronal brain slices stained using DAPI (*blue*) and an antibody specific for GFAP (*red*). In control GFP brain slices, GFAP^+^ GFP^+^ cells are highly dispersed within tumor mass in both hemispheres and show an elongated morphology suggesting a migratory behavior. DUSP8-GFP coronal slices show a reduction in GFAP^+^ GFP^+^ cells which are further reduced in sh-DUSP8-GFP. Interestingly, sh-DUSP8-GFP brain slices show GFAP^+^ GFP^−^ cells indicating that tumor cells appear surrounded and limited by endogenous mouse glial cells. Scale bar: 500 μm. Insets are higher magnification images confirming a different morphology and distribution of GFAP^+^ GFP^+^ and GFAP^+^ GFP^−^ in the different animal groups analyzed. Scale bar: 50 μm

Intracerebral injection of patient-derived GSCs generates highly infiltrative tumors that closely mimic the histology of malignant glioma [11, 35]. Stable GFP control vector-, DUSP8-GFP- or sh-DUSP8-GFP-expressing GSC#1 were grafted into the striatum of NOD-SCID mice. At 12 weeks after grafting, control (GFP) mice (*n* = 3) grafted with GFP GSC#1 cells harbored tumors that invaded the injected striatum, the tumor xenograft showed an expansive growth, thereby our method was highly reliable in assessing the tumor volume (Fig. 6A, *upper panel*). Moreover, from the injected striatum, tumor cells spread following the white matter paths, like the corpus callosum, optic tract, and anterior commissure, and cerebrospinal fluid (CSF) pathways, like ventricles and subarachnoid spaces (Fig. 6A). GFP^+^ cells appear more abundant and better organized in nodules in mice injected with GFP while DUSP8-GFP and sh-DUSP8-GFP brain slices showed a reduced number of GFP^+^ cells. Particularly, GFP^+^ cells appear less abundant in sh-DUSP8-GFP mice, showing a less elongated phenotype but rather a round morphology as compared with cells in control GFP brain slices, although they appear more disseminated and capable of reaching the controlateral hemisphere (Fig. 6A).

Analysis of tumor volumes in the striatum showed that mice injected with DUSP8-GFP- or sh-DUSP8-GFP-expressing GSC#1 cells harbored significantly smaller tumors than mice injected with GFP expressing GSC#1 cells (Fig. 6A, *upper panel*). The volume of the brain invaded by tumor cells at 12 weeks after grafting was 26.9 ± 0.8 mm^3^, 4.9 ± 1.2 mm^3^ and 1.8 ± 0.1 mm^3^ in mice grafted with GFP, DUSP8-GFP and sh-DUSP8-GFP GSC#1, respectively (mean ± SD, *p* = 0.002, DUSP8-GFP *vs* GFP; *p* = 0.0005, sh-DUSP8-GFP *vs* GFP, Student-*t* test).

At 16 weeks after grafting the volume of the brain invaded by tumor cells was 39.9 ± 1.2 mm^3^, 15.1 ± 0.6 mm^3^ and 10.5 ± 0.7 mm^3^ in mice grafted with GFP, DUSP8 GFP and sh-DUSP8 GFP GSC#1, respectively (mean ± SD, *p* = 0.001, DUSP8-GFP *vs* GFP; *p* = 0.001, sh-DUSP8-GFP *vs* GFP, Student-*t* test) (Fig. 6A, *lower panel*).

The reduction of tumor volume was confirmed by Ki67 labeling (Fig. 6B) identifying a decreased number of proliferating cells in DUSP8-GFP tumors further reduced in sh-DUSP8-GFP, as compared with GFP GSC#1 cells.

Staining for the vascular progenitor endothelial marker, CD34 [18], revealed the presence of double-positive GFP^+^ CD34^+^ cells in both GFP and sh-DUSP8-GFP GSC#1-derived brain tumors, the latter in almost all tumor cells, while double-positive cells were barely detectable in DUSP8-GFP tumor cells, confirming that DUSP8 silencing promotes the acquisition of the molecular determinants for endothelial transdifferentiation (Fig. 6C).

Moreover, as expected GFAP staining (Fig. 6D) revealed the presence of double positive, GFP^+^ GFAP^+^ cells, within both the GFP and DUSP8-GFP GSC#1 tumors. Interestingly, double positive cells were scarcely detectable in sh-DUSP8-GFP tumor where instead tumor cells resulted surrounded by mouse reactive glia cells, GFAP^+^ GFP^−^ cells, suggesting a strong reaction of mouse brain parenchyma. GFP cells in sh-DUSP8-GFP generated tumor showed a round shape morphology suggesting a reduced capacity to acquire a glial phenotype as confirmed by negative labeling for GFAP. Noteworthy, the presence of murine reactive glia surrounding and infiltrating sh-DUSP8-GFP tumor nodules suggests the ability of these cells to recruit endogenous reactive cells, likely mediated by releasing soluble molecules able to influence the tumor microenvironment (TME), in line with in vitro results.

Analysis of tumor cell proliferation, as assessed by Ki67 labeling index, showed that as compared with GFP expressing tumors, (42.7 ± 6.4, mean ± SD), those grafted with either DUSP8-GFP, sh-DUSP8-GFP GSC#1 cells showed a significantly reduced proliferation in sh-DUSP8-GFP tumors, (21.5 ± 4.1, mean ± SD) and even more reduced in DUSP8-GFP tumors (2.8 ± 1.3, mean ± SD) (Supplementary Fig. S9A-B).

Moreover, MVD analysis of brain tumors showed that microvessel counts (using CD31 staining) in sh-DUSP8-GFP (0.051 ± 0.01, mean ± SD), were significantly higher respect to either GFP (0.067 ± 0.006, mean ± SD), and DUSP8-GFP tumors (0.00026 ± 0.00042, mean ± SD) (Supplementary Fig. S9C).

Taken together, in vivo results confirm a crucial role for DUSP8 in GBM progression. Particularly, stable enforced expression of DUSP8 produces glial tumors with reduced infiltrating ability and very low frequency of proliferating cells and MVD, whereas, stable DUSP8 silencing gives raise to very small nodules of endothelial-like GFAP^low/−^ tumor cells, characterized by a very high MVD.

## Discussion

The inverse correlation between DUSP8 and miR-1825 expression and the significant association of DUSP8 down-regulation to higher MVD and poor OS of GBM patients, suggested a crucial role for DUSP8 in the GSC-associated neovascularization process. To confirm this hypothesis, we evaluated the effects of DUSP8 modulation on the tumorigenic ability of GSCs in vitro and in vivo. As expected, the enforced expression of DUSP8 impairs the ability of GdECs to form tube-like structures, confirming that the shutdown of DUSP8 phosphatase activity is mandatory to both morphological and functional transdifferentiation of GSCs into GdECs. Moreover, DUSP8 restoration increased sensitivity of GdEC#1 to ralimetinib treatment whereas GdEC#1 sh-DUSP8-GFP showed a decreased sensitivity confirming the critical role of DUSP8 in determining sensitivity to cancer therapeutics [36]. Surprisingly, both stable enforced expression and silencing of DUSP8 in GSC#1 dramatically reduced tumor growth following intracerebral injection into the striatum of NOD-SCID mice. This effect might be due to the fact that DUSP8 works as an “on/off” switch. In the early stages of tumor development, DUSP8 is in the “on” state, promoting proliferation while maintaining the undifferentiated characteristics of stem-like cells. On the contrary, when the tumor nodule has reached a certain limit size and requires oxygen and nutrients, the DUSP8 switch goes into “off” mode and the tumor cells begin to contribute to the formation of blood vessels and to migrate, infiltrating the surrounding tissue.

In the attempt to transpose the laboratory data to the clinical condition, we postulate that the stem-like fraction of tumor cells might differentiate towards the endothelial phenotype in tumor regions where the blood–brain-barrier is disrupted and a free passage of serum and of serum-related products occurs. Under such circumstances, the DUSP8 machinery is switched off, and the endothelial trans-differentiation of the GSCs sustains neovasculature.

Gene expression variations induced by DUSP8 modulation in GdEC#1, identified a list of 17 genes whose expression was regulated in opposite way in DUSP8-GFP GdEC#1 *vs* sh-DUSP8-GFP GdEC#1. These transcripts revealed the modulation of genes associated with EMT pathway, confirming that the shutdown of DUSP8 phosphatase activity and, therefore, the activation of MAPK pathway, is mandatory to endothelial transdifferentiation. Interestingly, among the transcripts down-regulated in DUSP8-GFP GdEC#1 and up-regulated in sh-DUSP8-GFP GdEC#1 we found POSTN, a matricellular protein that has been associated with glioma progression, particularly with glioma angiogenesis [34]. We recently demonstrated that POSTN is an important mediator of the effects of endothelial cell-derived extracellular vesicles on tumorigenic properties of GSCs [15]. Noteworthy, among the transcripts down-regulated in DUSP8-GFP GSC#1 and up-regulated in sh-DUSP8-GFP GSC#1 we found STC1, a hormone-like protein that exists in the extracellular matrix which contributes to the malignant progression of GBM and is associated with resistance to TMZ therapy [33].

Analysis of supernatants revealed that DUSP8 modulation exerts opposite effects in the release of four key molecules implicated in GBM. In the GSC context, DUSP8 oppositely regulates CX3CL1 and TNF-RII secreted molecules, both related with disease severity [37, 38]. As regards GdECs, we found that DUSP8 modulation affects the secretion of TIMP-1 and IL-4 soluble molecules in an opposite way. TIMP-1 was one of the preferentially up-regulated genes in IDH-wild type gliomas and its higher expression indicated worse prognosis of glioma patients [39]. The role of IL-4 signalling in multiple cancer types, including GBM, is widely documented [40]. It has been shown that blocking autocrine and paracrine IL-4 signalling impairs breast cancer stem-like cell proliferation, invasion, and tumor growth by downregulating MAPK pathway activity through up-regulation of DUSP4 [41].

Altogether our data confirms a major role for DUSPs not only in the regulatory mechanisms of intracellular signaling pathways in a variety of biological processes but, additional functions that push to target DUSPs with potential therapeutic modalities. However, the complex and unique, yet contradictory, roles that DUSPs play in various cancers, exhibiting both tumor suppressor and tumor promoter activity in different cancers, require special caution.

Most of the DUSPs that have thus far been implicated in brain cancers were identified in GBM. Lin and colleagues, showed that dexamethasone treatment inhibited MMP-2 mediated invasiveness of human malignant glioma cells through a DUSP1-dependent mechanism, implicating the tumor suppressor characteristics of DUSP1 [42], as well as, DUSP4 and DUSP26 whose down-regulation correlates with invasive phenotypes of GBM [43, 44]. Conversely, DUSP6 has tumor-promoting properties in GBM, as evidenced by the transcriptional up-regulation of DUSP6 in primary and long-term cultures of GBM [45].

On the other hand, the development of pharmacological DUSP inhibitors for clinical use is currently in progress, many of which work by inducing the formation of ROS. By screening more than 300 compounds, we recently selected elesclomol to be effective on GSCs and on GdECs as well. In depth investigation of the molecular mechanisms underlying GSC and GdEC response to elesclomol, confirmed that this compound induces a strong increase in mitochondrial ROS in both GSCs and GdECs ultimately leading to a non-apoptotic copper-dependent cell death [14]. These findings provide a strong rationale to develop therapeutic strategies based on modulation of DUSP8 for GBM treatment.

## Supplementary Information

Supplementary Material 1.

## Data Availability

The datasets used and/or analyzed during the current study are available from the corresponding author upon reasonable request.

## Abbreviations

CD133: Prominin 1
CD31: Platelet and endothelial cell adhesion molecule 1
CD34: CD34 molecule
CSF: Cerebrospinal fluid
CX3CL1: Fractalkine
CX3CR1: CX3C motif chemokine receptor 1
CXCL10: C-X-C motif chemokine ligand 10
DUSP: Dual-specificity phosphatase
ECM: Extracellular matrix
EMT: Epithelial to mesenchymal transition
ERK: Extracellular signal-regulated kinase
FDA: Food and Drug Administration
GBM: Glioblastoma
GdEC: GSC-derived endothelial cell
GFAP: Glial fibrillary acidic protein
GSC: Glioblastoma stem-like cell
GSEA: Gene set enrichment analysis
ICAM-1: Intercellular adhesion molecule 1
IDH: Isocitrate dehydrogenase
IFN-γ: Interferon-γ
IL-4: Interleukin-4
JNK: C-Jun N-terminal kinase
Ki67: Marker of proliferation Ki-67
MAPK: Mitogen-activated protein kinases
MGMT: O-6-methylguanine-DNA methyltransferase
MiRNA: Microrna
MMP: Metalloproteinase
MVD: Microvascular density
OS: Overall survival
p38: Mitogen-activated protein kinase p38
PFS: Progression-free survival
POSTN: Periostin
ROS: Reactive oxygen species
STC-1: Stanniocalcin-1
sTNF-R: Soluble tumor necrosis factor receptor
Tie2: TEK Receptor Tyrosine Kinase
TIMP-1: Tissue inhibitor of metalloprotease-1
TME: Tumor microenvironment
TMZ: Temozolomide
TNF RII: Tumor necrosis factor receptor II
TNF-α: Tumor necrosis factor-α
VCAM-1: Vascular cell adhesion molecule 1
VEGF: Vascular endothelial growth factor
VEGFR/KDR: Vascular endothelial growth factor receptor /kinase insert domain receptor
VPs: Vascular progenitor cells
WHO: World Health Organization
