# Supplementary material for: DUSP8 as a regulator of glioblastoma stem-like cell contribution to tumor vascularization
Source: J Exp Clin Cancer Res. 2025 Sep 30;44:269. doi: 10.1186/s13046-025-03515-3 (PMC12486788; doi:10.1186/s13046-025-03515-3)
Supplement: Supplementary file 1 — Supplementary Material 1. [file 13046_2025_3515_MOESM1_ESM.pdf]

Cell cultures

After mechanical dissociation of GBM sample, the resultant cell suspension was maintained in stem cell serum-free medium supplemented with 10 ng/ml basic fibroblast growth factor (b-FGF) (PeproTech, London, UK) and 20 ng/ml epidermal growth factor (EGF) (PeproTech), as previously described (1, 2). To validate GSC lines, Short Tandem Repeat (STR) DNA fingerprinting was performed. Nine highly polymorphic STR loci plus amelogenin (Cell IDTM System, Promega Inc., Madison, WI, USA) were used. Samples were amplified and electrophoretically separated on an ABI Prism 3100 Genetic Analyzer (Applied Biosystems™ by ThermoFisher, Waltham, MA, USA). Data analysis was performed by GeneMapper® software, version 3.2 (Applied Biosystems by ThermoFisher). All GSC profiles were challenged against public databases to confirm authenticity (3).

The cervical cancer HeLa cells and the human 293T embryonic kidney cells, were purchased from the ATCC. Both cell lines were maintained in DMEM (Euroclone, Pero, MI, Italy) supplemented with 10% (v/v) heat-inactivated FBS (Euroclone), 2 mM L-glutamine, 100 U/ml of penicilline and 100 μg/ml of streptomycin (Euroclone).

All cell lines were regularly checked to exclude mycoplasma contamination by Mycoalert® Detection Kit (Lonza Walkersville Inc., Walkerswille, MD, USA).

Transdifferentiation of GSCs

GSCs were maintained in stem cell medium supplemented with EGM™-2MV SingleQuots™ Kit (Lonza Walkersville Inc.) and 12 μg/ml Bovine Brain Extract (BBE, Lonza Walkersville Inc.) on Matrigel® (Corning, New York, NY, USA) coated tissue culture surface under hypoxic condition (1% O_2) for two weeks in the InvivO2 Physiological Cell Culture Workstations (The Baker Company, Inc., Sanford, ME, USA). Under these conditions, GSCs grow as continuous net-like structures.

Flow cytometry

Cells were incubated with the antibodies for 90 minutes at 4°C, washed with PBS and analyzed by CytoFLEX LX flow cytometer (Beckman Coulter, Brea, CA, USA) equipped with a CytExpert software (Beckman Coulter Life Science, Milan, Italy). The antibodies used were as follows: phycoerythrin (PE)-conjugated mouse anti-human CD31 antibody (1:20, BD Biosciences, Milan, Italy); PE-conjugated mouse anti-human CD34 antibody (1:20, clone BIRMA-K3, DakoCytomation, Denmark); PE-conjugated mouse anti-human CD133/1 antibody (1:20, clone AC133, Miltenyi Biotec Inc., Bergisch Gladbach, Germany); PE-conjugated mouse anti-human Tie2 antibody (1:25, R&D Systems, Minneapolis, MN, USA); PE-conjugated mouse anti-human VEGFR2 (KDR) antibody (1:25, R&D Systems) or PE-conjugated mouse IgG_1 isotype control antibody (Miltenyi Biotec Inc.). Data were analyzed with CytExpert software (Beckman Coulter).

RT-PCR

To detect the expression levels of miR-1825 (miRBase ID MIMAT0006765), miR-1281 (miRBase ID MIMAT0005939), and miR-4516 (miRBase ID MIMAT0019053), RT-PCR was carried out using TaqMan® Advanced MicroRNA Assays protocol (Applied Biosystems™ by ThermoFisher Scientific) and normalized with RNU6B (assay ID 001093, Applied Biosystems™ by ThermoFisher Scientific). RT-PCR for DUSP8 mRNA detection was performed with SYBR™ Green Master Mix (Applied Biosystems™ by ThermoFisher Scientific) and normalized with the housekeeping gene, GAPDH. All reactions were run in duplicate in the QuantStudio 12K Flex (Applied Biosystems™ by ThermoFisher Scientific). The specific primers were listed in the following table:

	**For: 5’- > 3’**	**Rev: 3’- > 5’**
**GAPDH**	ACCTGACCTGCCGTCTAG	CCTGCTTCACCACCTTCT
**DUSP8**	TCCCGAGGAAGGTGATGGAT	AGCTTGGAGCAGCAGATGTT

Automated capillary Western immunoassay (WES)

The following primary antibodies were used: DUSP8 (NBP2-92392) and β-tubulin (NB600-936) from Novus Biologicals by Bio-Techne, p38 (#9212), P-p38 (#4511), P-ERK (#9101), JNK (#9252)

from Cell Signaling (Danvers, MA, USA), P-JNK (AF1205) from R&D Systems by Bio-Techne, ERK (sc-514302) from Santa Cruz Biotechnology (Dallas, TX, USA), β-actin (A5441) from Sigma-Aldrich (St. Louis, MO, USA).

Luciferase Reporter Assay

The two human DUSP8 3′-UTR sequences containing the target sites for miR-1281, miR-1825 and miR-4516 (DUSP8 3'-UTR nucleotides (nt) 396-940 and DUSP8 3'-UTR nt 1461-2190), were cloned into psiCHECK^{TM}-2 Vector (Promega Inc.) downstream of the Renilla luciferase reporter gene. The mutant plasmids containing mutations of the miR-1825 target sites were obtained by site-specific mutagenesis. All the plasmids were verified by sequence analysis. 293T cells were transiently co-transfected by Lipofectamine 2000 (Invitrogen by ThermoFisher Scientific), with 400 ng of luciferase reporter plasmid containing wild-type (wt) or mutated (mut) DUSP8 3′-UTR-seed sequences, 10 pmol of either the hsa-miR-1281, hsa-miR-1825 and hsa-miR-4516 mimic or control-mimic oligonucleotides (Ambion, Life technologies by ThermoFisher Scientific). 36 hours post-transfection, cells were lysated to measure the Renilla and Firefly luciferase activities using Dual-luciferase Reporter Assay Kit (Promega Inc.) according to the manufacturer's instructions.

RNA Immunoprecipitation (RIP)

HeLa were plated at a density of $2x10^5$ cells/ml and transiently transfected with 400 pmol of scrambled or hsa-miR-1825 mimic by Lipofectamine 2000 (Invitrogen by ThermoFisher Scientific). Cells were lysed 24 hours post-transfection, and the obtained lysates were incubated with protein G magnetic beads and a pan-Ago antibody that recognizes Ago 1/2/3. Then, precipitated RNAs were purified, and RT-PCR was performed to detect the levels of DUSP8, using the primers reported above.

Analysis of conditioned medium by Luminex assay

In addition to classic cytokines and chemokines with inflammatory activity, we selected several molecules and growth factors related to adhesion/invasion and angiogenic abilities. Quantification of interferon-γ (IFN-γ), interleukin(IL)-1 β, IL-2, IL-4, IL-6, IL-8 (CXCL8), IL-10, IL-12, IL-17, Eotaxin (CCL11), monocyte chemoattractant protein-1 (MCP-1 or CCL2), macrophage Inflammatory Protein(MIP)-1α (CCL3), MIP-1β (CCL4), fractalkine (CX3CL1), growth regulated protein alpha (GRO-α or CXCL1), interferon-γ-induced protein 10 (IP-10 or CXCL10), tumor necrosis factor α (TNF-α), soluble TNF receptor superfamily member 8 (TNFRSF8 or CD30), soluble TNF receptor 1 (TNF RI or TNFRSF1A), soluble TNF RII (TNFRSF1B), epithelial growth factor (EGF), basic fibroblast growth factor (bFGF or FGF2), granulocyte-colony stimulating factor (G-CSF), granulocyte-macrophage colony-stimulating factor (GM-CSF), platelet-derived growth factor-BB (PDGF-BB), VEGF, intercellular adhesion molecule 1 (ICAM-1 or CD54), vascular cell adhesion molecule 1 (VCAM-1 or CD106), matrix metalloproteinase(MMP)-2, MMP-7, MMP-9, MMP-10, tissue inhibitor of metalloprotease-1 (TIMP-1), was performed using customized assay (Human Luminex® Discovery Assay, R&D Systems, Minneapolis, MN) according to the manufacturer's instructions. Data were acquired on the Bio-Rad Bio-Plex X200 reader and analyzed using Bio-Plex Software Manager™ 6.1 (Bio-Rad, Hercules, CA, USA). The concentration of analytes was expressed in pg/10^6 cells or ng/10^6 cells.

Microarrays and RNA-Sequencing analysis (RNA-Seq)

MiRBase (http://mirbase.org/) algorithm was used for miRNA target prediction and gene set enrichment analysis (GSEA) was based on MSigDB using the GSEA online tool hosted by the Broad Institute (http://www.broadinstitute.org/gsea/index.jsp).

RNA-Seq libraries were prepared according to the manufacturer's instructions. Libraries were sequenced in paired-ends on an Illumina NovaSeq 6000 System (Illumina, San Diego, CA, USA).

The samples were mapped on reference genome (https://www.gencodegenes.org/human/) using the bioinformatics tool STAR (version 2.7.10b) (4), with the standard parameters for paired reads. The

reference track was the Human assembly obtained from GenCode (HG38 - Release 35 v44 (GRCh38.p14). The quantification of transcripts expressed for each sequenced samples was performed using featureCount algorithm (5).

To identify differentially expressed genes in DUSP8- and sh-DUSP8-GFP transduced cells compared to control vector GFP, "R" was used to create a matrix of all transcripts expressed in all samples with the corresponding read-counts. The Bioconductor package NOISeq (6, 7) was used to normalize the data, using RSEM method and then to perform the differential expression analysis.

Tube formation assay

The ability to form tube-like structures in GFP, DUSP8-GFP or sh-DUSP8-GFP GdECs, or in GdECs after ralimetinib treatment was evaluated by tubule formation assay. Briefly, a 48-well plate was coated with 135 µl of Matrigel® per well, and then GdECs were seeded in the Matrigel®-coated 48-well plate at a density of 3×10^4 cells per well. After 6 h, imaging acquisition was performed by Axiovert A1 KMAT Zeiss microscope (Zeiss Italy, Milan, Italy). The total tube length in four random microscopic fields was measured by using microscope software analysis (Zeiss Zen 3.6).

Intracranial implantation of GSCs into immunocompromised mice

GFP, DUSP8-GFP and sh-DUSP8-GFP expressing GSC#1 line were resuspended in 5 µl of stem cell serum-free DMEM. For brain grafting, the mice were anesthetized with intraperitoneal injection of diazepam (2 mg/100 g) followed by intramuscular injection of ketamine (4 mg/100 g). Animal skulls were immobilized in a stereotactic head frame and a burr hole was made 2 mm right of the midline and 1 mm posterior to the coronal suture, and cells were slowly injected using the tip of a 10-µl Hamilton microsyringe placed at a depth of 3 mm from the dura.

After 12 and 16 weeks from grafting, animal were deeply anesthetized and transcardially perfused with 0.1 M PBS (pH = 7.4), followed by 4% paraformaldehyde in 0.1 M PBS

Immunofluorescence analysis of tumors in brain slices

After perfusion, the brains were removed from the skull of mice and post-fixed by 4% paraformaldehyde in 0.1 M PBS solution, overnight at 4°C. After rinsing in phosphate buffer, brains were cryoprotected by sequential incubation with 15% sucrose and then 30% sucrose solutions, both overnight at 4°C. Free-floating brain (25 □m thickness) for the different experimental groups (n = 6) were analyzed for the presence and distribution of GFP^+ GSCs. Images of each brain slice was acquired using a 10X or a 20/0.8NA objective on a Leica THUNDER Imager DMI8 fluorescence microscope (Leica Microsystems, Wetzlar, Germany). Cellular fields within brain slices were imaged using a dry 20/0.8NA or 40X objective. Image z-stacks with a z-step size of 0.5 µm were acquired to ensure adequate sampling of different fields of the analyzed brain slices. A tile scan function was used to rapidly image the entire brain slice using the Leica LASX software.

Free-floating sections were then analyzed for immunofluorescence, while they were kept for long storage at −20°C in freezing solution (containing ethylene glycol and glycerol). After rinsing in PBS, sections were incubated in 10% normal horse serum in phosphate buffer containing 0.2% Triton X-100 for 30 minutes to reduce non-specific binding. All sections were labelled with DAPI for nuclei segmentation (excitation wavelength used for imaging 391 nm) and anti-GFAP (clone EP672Y) rabbit monoclonal (Sigma-Aldrich), or anti-Ki67 (clone 30-9) rabbit monoclonal (Roche, Rotkreuz, Switzerland) or anti- CD34 (clone QBEnd 10) mouse monoclonal (Dako), diluted in phosphate buffer containing 2% normal horse serum and 0.2% Tritox X-100, overnight at 4°C. After rinsing, the sections were incubated with labelled secondary antibodies donkey anti-mouse 647 or donkey anti-rabbit 594 (Alexa Fluor Molecular Probes) for 1 hour at RT. After a thorough rinse, the sections were incubated in phosphate buffer containing a Hoechst for 10 minutes at RT; sections were mounted on slides and coverslipped with antifade medium (ProLong Glass mountant, Invitrogen).

The images were subjected to small volume computational clearing (SVCC) using LASX software. The cranio-caudal extension of the brain area invaded by GFP^+ GSCs was assessed on serial coronal sections. The volume of the brain invaded by the tumor was determined according to the equation,

V = *A x b*, where *A* is the mean of the tumor area calculated on coronal sections through the ImageJ software, Fiji (8) and *b* is the cranio-caudal extension of the tumor.

Immunohistochemistry, microvessel density evaluation (MVD) and combined in situ Hybridization/Immunohistochemistry (ISH/IHC)

Briefly, 4-μm thick sections were obtained from formalin-fixed paraffin-embedded (FFPE) blocks and mounted on silanized slides. For antigen retrieval, deparaffinized and rehydrated sections were treated with citric acid buffer (pH 6.0) for three cycles of 5 minutes each in a 750 W microwave oven, followed by inhibition of endogenous peroxidase with 3% H_2O_2 for 10 minutes at room temperature. The sections were then incubated with a pre-diluted rabbit anti-DUSP8 polyclonal antibody (NOVUS biologicals, Colorado, USA) or anti-GFP (clone 168AT1211) mouse monoclonal (Abcepta, Inc. San Diego CA) or anti-GFAP (clone EP672Y) rabbit monoclonal (Sigma-Aldrich) or anti-Ki67 (clone 30-9) rabbit monoclonal (Roche) or anti-CD31 (Clone JC70) mouse monoclonal antibody (Roche). The primary antibody was visualized using the avidin–biotin–peroxidase complex method (UltraTek HRP Anti-polyvalent; ScyTek, Logan, Utah, USA), according to the manufacturer's instructions. 3,3'-Diaminobenzidine tetrahydrochloride was used as the enzyme substrate to observe the specific antibody localization, and Mayer's hematoxylin was used as a nuclear counterstain (9). Human colorectal cancer was used as positive control. Negative controls were obtained by replacing the primary antibody with a non-immune immunoglobulin of the same isotype or phosphate-buffered saline solution (PBS, pH 7.4). The results of IHC reactions were independently evaluated by two pathologists (MM and GR) who were blinded to the clinicopathologic data. Variations in enumeration, within a range of 5%, were re-evaluated on a consensus basis using a double-headed microscope.

To evaluate DUSP8 expression, a combined score based on the percentage of positive cells and staining intensity was used. The score for the extent of the IHC-stained area was scaled as 0 for no IHC signal, 1 for 1–30%, 2 for 31–70%, and 3 for 71–100% of tumor cells stained. The score for IHC

intensity was also scaled as 0 for no IHC signal, 1 for weak, 2 for moderate, and 3 for strong IHC signals. The final score (ID-score) used in the analysis was calculated by multiplying the extent and intensity scores, with a maximum score equal to 9; the ID scores 0–3 and 4–9 discriminate samples with low or high expression, respectively.

MVD of GBMs (n = 24) was measured according to the method previously described with few modifications (9). Briefly, in areas of most intense neovascularization, individual microvessel counts (using CD31 staining) were made on a 200X magnification field (equivalent to 0.7386 mm^2). Any endothelial cell or endothelial cell cluster was considered as a single countable microvessel. MVD was expressed as the mean number of microvessels per field from 3 highly vascularized areas in each case.

For combined ISH/IHC, 5-μm thick sections were obtained from FFPE blocks and mounted on silanized slides, then incubated at 60°C for 1 hour. The IHC procedure for DUSP8 was performed as reported above, with the following modifications: hematoxylin counterstaining was omitted, and the slides were not mounted with coverslips to allow subsequent chromogenic in situ hybridization (CISH) analysis. For the detection of miR-1825, a CISH assay was performed on the same slides, using miRCURY LNA miRNA detection probes (QIAGEN, Milan, Italy). Proteinase K treatment was applied at 37°C for 10 minutes in a hybridization chamber, followed by PBS washes. Briefly, after dilution with hybridization buffer, tissue slides were incubated with miR-1825 detection probe at 50°C for 1 hour in a hybridization chamber, following the manufacturer’s protocol. Post-hybridization washes were performed using SSC buffer and PBS. Detection was carried out using an Anti-DIG antibody, diluted 1:800, incubated for 1 hour at RT, and chromogenically developed with BCIP/NBT and levamisole. After the CISH procedure, the slides were mounted with Eukitt medium. Coverslips were applied, and the slides were left to dry overnight before microscopic evaluation. Human heathy brain tissue and colorectal cancer were used as positive control for miR-1825 and DUSP8, respectively. Negative controls were obtained by replacing the primary antibody or probe with PBS.

Tissue sections were examined under a light microscope to assess the correlation between DUSP8 expression and miR-1825 signals. DUSP8 expression, brown staining, was evaluated based on the localization (mainly cytoplasm), intensity and percentage of positive cells. MiR-1825 expression was detected as nuclear and cytoplasmatic blue staining, evaluating the intensity and percentage of positive cells. The evaluation aimed to determine whether a correlation existed between the expression levels of DUSP8 and miR-1825 within the same tissue sections. The correlation was assessed by comparing the intensity of staining for both molecules in corresponding regions of the tissue across multiple fields.

GBM patient cohort

This study includes 50 consecutive adult IDH-wildtype GBM patients who underwent craniotomy for resection of histologically confirmed GBM (World Health Organization grade 4) in the supratentorial compartment, and who were homogeneously treated with radiotherapy with concomitant and adjuvant temozolomide (TMZ) at the Fondazione Policlinico Universitario A. Gemelli IRCCS, Rome, Italy. Ethical approval was obtained by the local Ethics Committee (Prot. ID 2253 and 3782) and all patients provided written informed consent. Patients of pediatric age (under 18 years) were not included in this study. Clinical features of GBM patients and tumors are shown in Supplementary Table S2. The patients were aged 22 to 80 years at the time of primary surgery (median age, 60 years; mean age, 58.3 ± 11.6 years); 30 were men and 20 were women. The extent of tumor resection was evaluated on Gd-enhanced axial T1-weighted magnetic resonance imaging obtained 24-72 hours after surgery.

Statistical analysis

Statistical analysis was performed using GraphPad-Prism 5 software (GraphPad software, www.graphpad.com). Overall survival curves of patients with DUSP8 expression were estimated by Kaplan-Meier survival curves and differences in survival between groups of patients were compared

using the log-rank test. A statistical comparison of continuous variables between groups was performed using the Student's t-test, as appropriate. Categorical variables were compared using statistic chi-square and Fisher's exact test. Only values of $p<0.05$ were considered as significant and the level of significance is indicated in the plots using asterisks as follows: * for $p<0.05$, ** for $p<0.01$ and *** for $p<0.001$.

1. Ricci-Vitiani L, Pallini R, Larocca LM, Lombardi DG, Signore M, Pierconti F, et al. Mesenchymal differentiation of glioblastoma stem cells. Cell Death Differ. 2008;15(9):1491-8.

2. Pallini R, Ricci-Vitiani L, Banna GL, Signore M, Lombardi D, Todaro M, et al. Cancer stem cell analysis and clinical outcome in patients with glioblastoma multiforme. Clin Cancer Res. 2008;14(24):8205-12.

3. Visconti P, Parodi F, Parodi B, Casarino L, Romano P, Buccarelli M, et al. Short tandem repeat profiling for the authentication of cancer stem-like cells. Int J Cancer. 2021;148(6):1489-98.

4. Dobin A, Davis CA, Schlesinger F, Drenkow J, Zaleski C, Jha S, et al. STAR: ultrafast universal RNA-seq aligner. Bioinformatics. 2013;29(1):15-21.

5. Liao Y, Smyth GK, Shi W. featureCounts: an efficient general purpose program for assigning sequence reads to genomic features. Bioinformatics. 2014;30(7):923-30.

6. Tarazona S, Garcia-Alcalde F, Dopazo J, Ferrer A, Conesa A. Differential expression in RNA-seq: a matter of depth. Genome Res. 2011;21(12):2213-23.

7. Tarazona S, Furio-Tari P, Turra D, Pietro AD, Nueda MJ, Ferrer A, et al. Data quality aware analysis of differential expression in RNA-seq with NOISeq R/Bioc package. Nucleic Acids Res. 2015;43(21):e140.

8. Schindelin J, Arganda-Carreras I, Frise E, Kaynig V, Longair M, Pietzsch T, et al. Fiji: an open-source platform for biological-image analysis. Nat Methods. 2012;9(7):676-82.

9. Martini M, Cenci T, D'Alessandris GQ, Cesarini V, Cocomazzi A, Ricci-Vitiani L, et al. Epigenetic silencing of Id4 identifies a glioblastoma subgroup with a better prognosis as a consequence of an inhibition of angiogenesis. Cancer. 2013;119(5):1004-12.

Supplementary Table S1. Clinical features of GSC-generating GBM patients and tumors

Case Code	Age/ Sex	Sympt. (mo.)	Prim Rec	Location	MGMT	IDH1/2	PFS (mo.)	OS (mo.)
1	40/M	2.5	P	temporal	M	wt	6	12.5
61	59/M	2	P	occipital	UM	wt	3	6
83	52/M	0.5	P	temporal	UM	wt	4	8
163	56/M	5	P	parietal	UM	wt	1	2
275<br>275 BIS	58/M	2	P<br>R	occipital	M	wt	6	12
351	52/F	1	P	temporal	M	wt	84	84
412<br>486	56/F	1	P<br>R	frontal	UM	wt	22	31
450	76/F	1	P	temporal	M	wt	3	6

Age is displayed in years; Sympt., symptom duration; Prim., primary tumor; Rec., recurrent tumor; M/UM, methylated/unmethylated; PFS, progression-free survival; OS, overall survival.

Supplementary Table S2. Clinical features of GBM patient cohort

	**n = 50**
**Age, mean (±SD)**	59 (12.7)
**Age n (%)**	
**≤50**	14 (28%)
**>50**	36 (72%)
**Gender, n (%)**	
**Male**	31 (62%)
**Female**	19 (38%)
**Total Removal n (%)**	
**Yes**	24 (48%)
**No**	26 (52%)
**KPS n (%)**	
**≤70**	32 (64%)
**>70**	18 (36%)
**Ki67 expression n (%)**	
**<30**	20 (40%)
**≥30**	30 (60%)
**p53 expression n (%)**	
**≤15**	21 (42%)
**>15**	29 (58%)
**MGMT status n (%)**	
**Methylated**	24 (48%)
**Unmethylated**	26 (52%)
**DUSP8 expression n (%)**	
**≤3**	25 (50%)
**>3**	25 (50%)
**OS n (%)**	
**High**	22 (44%)
**Low**	28 (56%)

Age is displayed in years; OS, overall survival (mean value).

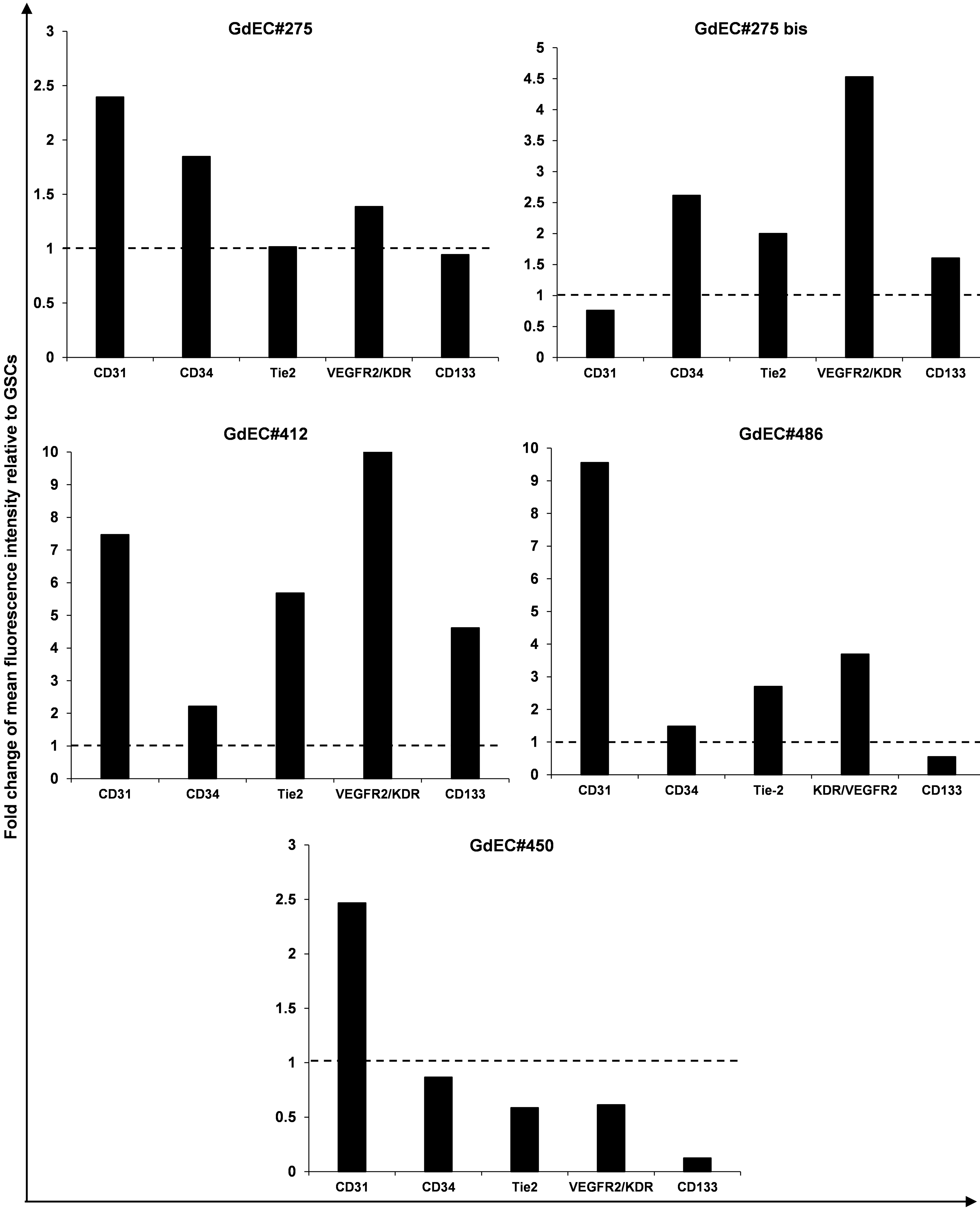

Supplementary Figure S1. Cytofluorimetric evaluation of CD31, CD34, Tie2, VEGFR2/KDR and CD133 endothelial marker expression in five patient-derived GdEC lines, after two weeks of culture in endothelial conditions. The baseline (dashed line at value 1) represents the expression of the same markers in the GSC counterpart, cultivated in stem cell conditions, used as reference.

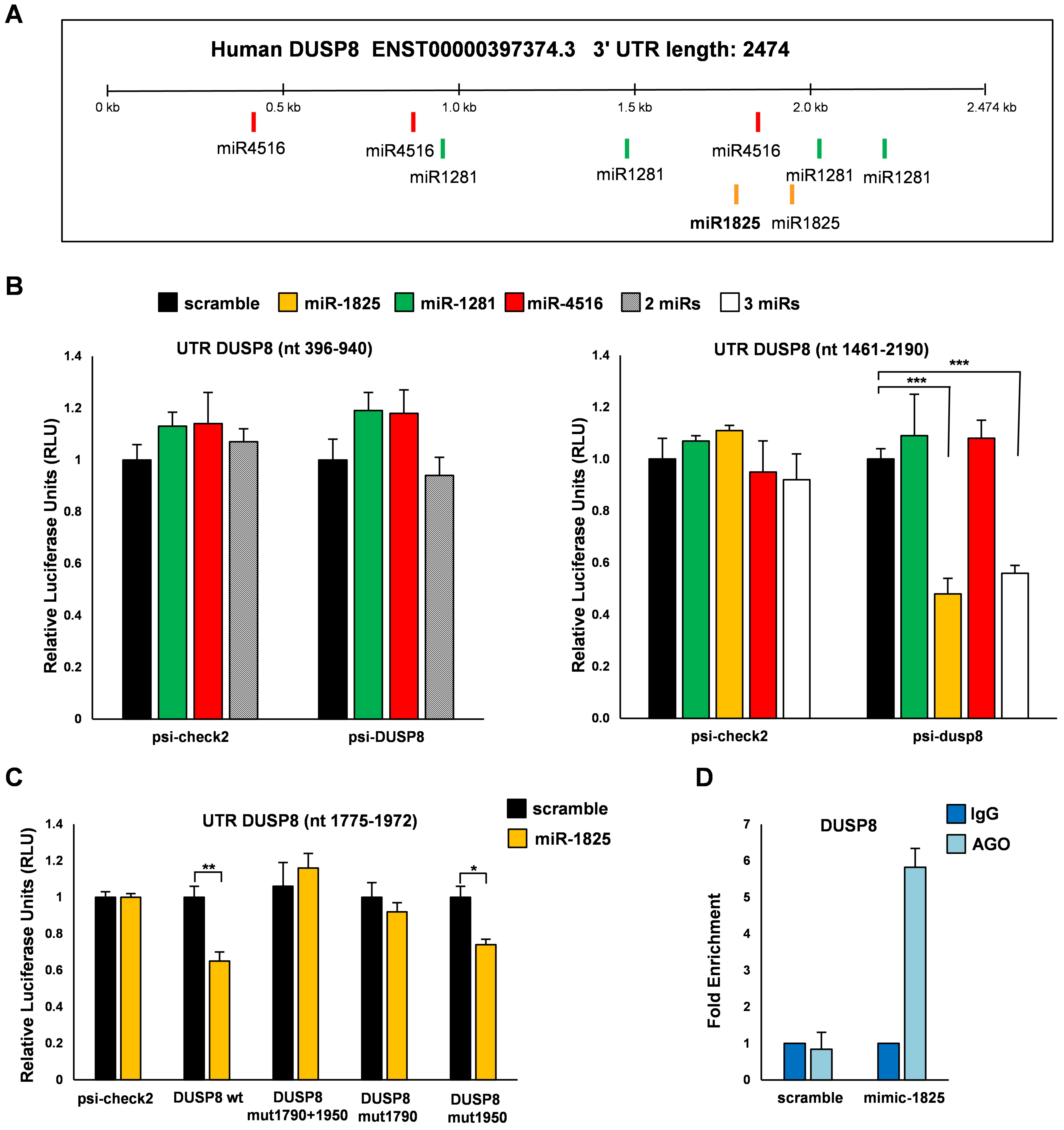

Supplementary Figure S2. A. Schematic representation of the target sites for miR-1281, miR-1825 and miR-4516 in the human DUSP8 3′-UTR. **B.** Dual-luciferase reporter assay in 293T transiently co-transfected with the three mimics and the reporter vectors containing different regions (nucleotides 396-940 and nucleotides 1461-2190) of the DUSP8 3’-UTR. **C.** Dual-luciferase reporter assay in 293T transiently co-transfected with miR-1825 mimic or scrambled and the reporter vectors containing the wild type (wt) or the mutated (mut) DUSP8 3’-UTR. Histograms show normalized mean values of the relative luciferase activity. Error bars represent the mean ± SD (n = 3). *** p<0.001, ***p*<0.01; **p*<0.05 (Student’s *t* test). **D.** RT-PCR on RNA recovered after RNA Immunoprecipitation assay with a pan-Ago and control IgG antibodies to pulldown the endogenous DUSP8 in HeLa transiently transfected with miR-1825 mimic or scrambled

A

	P	HR	95% CI of Exp(b)
Age (≤50 *vs* >50)	0.06	1.8152	0.9797 to 3.3634
Gender (Male *vs* Female)	0.64	0.8672	0.4784 to 1.5721
Total Removal (Yes *vs* No)	0.72	1.1127	0.6182 to 2.0028
KPS (≤70 *vs* >70)	0.0042	0.4105	0.2231 to 0.7554
Ki67 expression (<30 *vs* ≥30)	0.4247	0.7765	0.4173 to 1.4450
p53 expression (≤15 *vs* >15)	1.00	1.0008	0.5524 to 1.8132
MGMT status (methylated *vs* Unmethylated)	0.048	0.5410	0.2936 to 0.9948
DUSP8 expression (High *vs* Low)	0.0062	2.0751	1.3440 to 4.4453

B

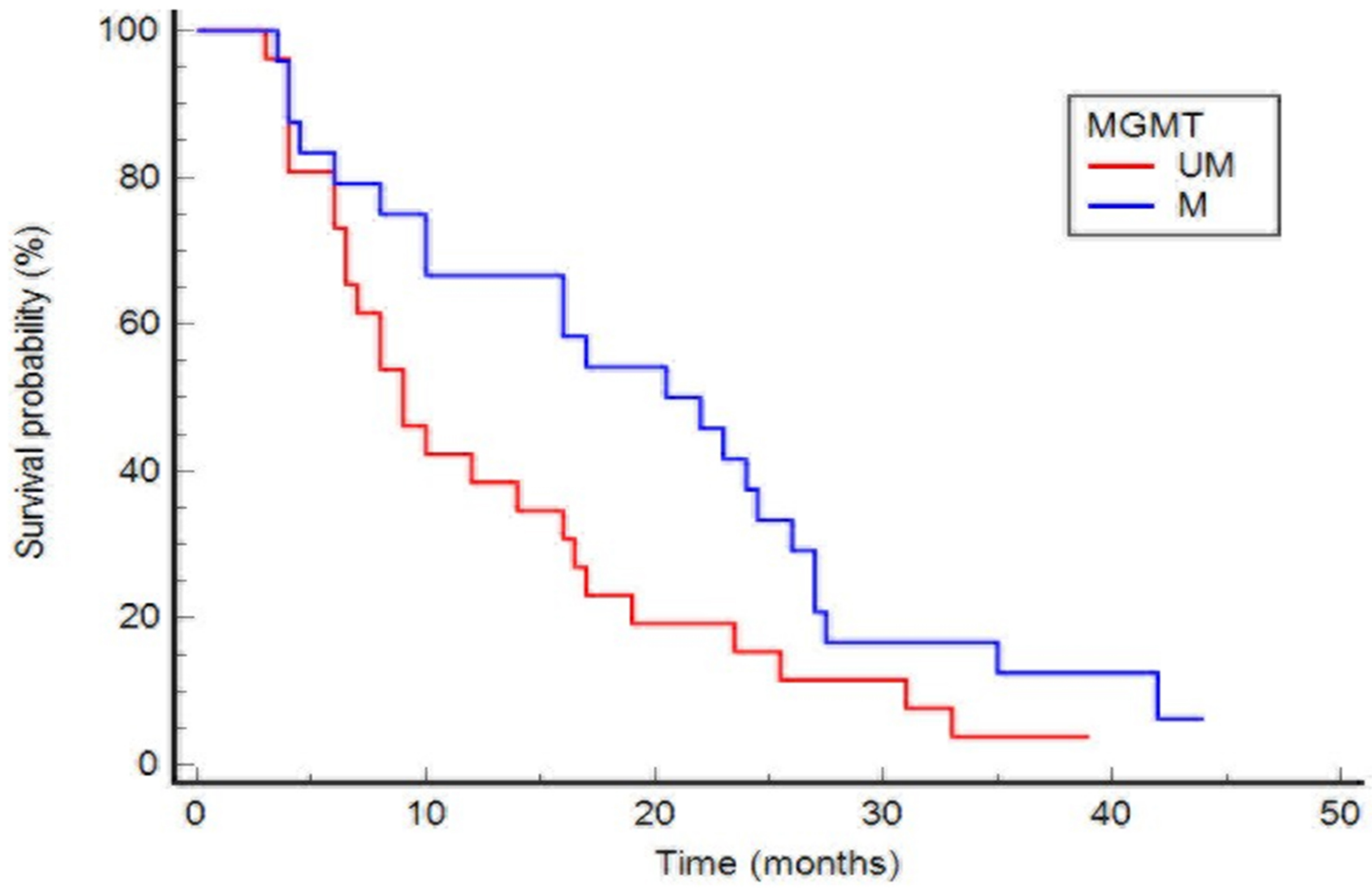

p=0.048, HR 0.54, 95% CI from 0.3 to 0.99

C

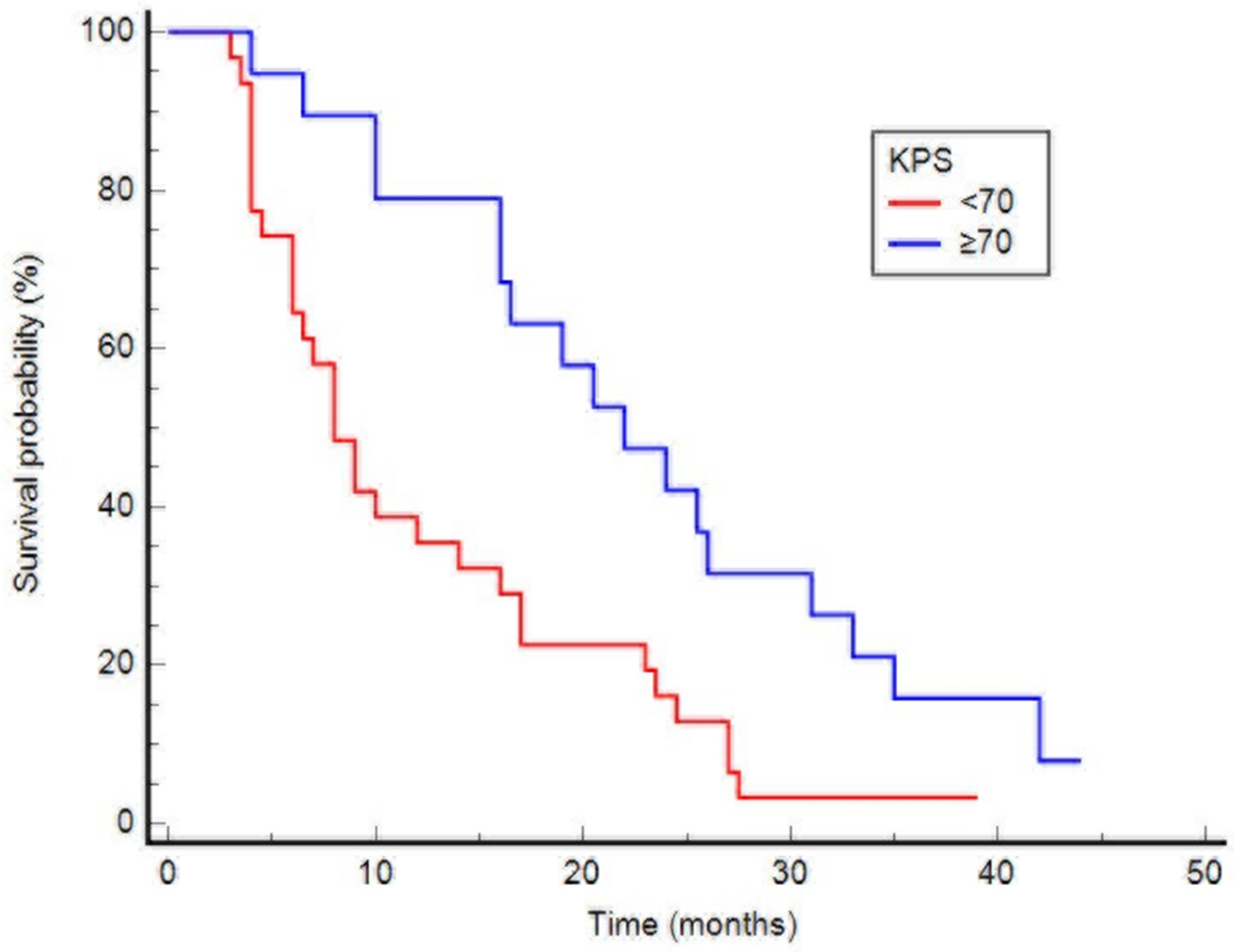

p=0.0042, HR 0.41, 95% CI from 0.22 to 0.75

D

Covariate	b	SE	Wald	P	Exp(b)	95% CI of Exp(b)
DUSP8	-0,7227	0,3205	5,0837	0,0242	0,4854	0,2590 to 0,9099
MGMT	-0,4486	0,3093	2,1029	0,1470	0,6385	0,3483 to 1,1708
KPS	-0,7805	0,3231	5,8379	0,0157	0,4582	0,2432 to 0,8630

Supplementary Figure S3. A. Univariate analysis for OS of the main clinical and biological GBM features. **B-C.** Kaplan-Meier curves for OS of MGMT **(B)** and KPS parameters **(C)**. Patients with MGMT methylation and higher KPS, were significantly associated with a better OS (p = 0.049 for MGMT status, p= 0.004 for KPS; *blue line*) in comparison to those with unmethylated MGMT and lower KPS (*red line*). **D.** Multivariate analysis for OS.

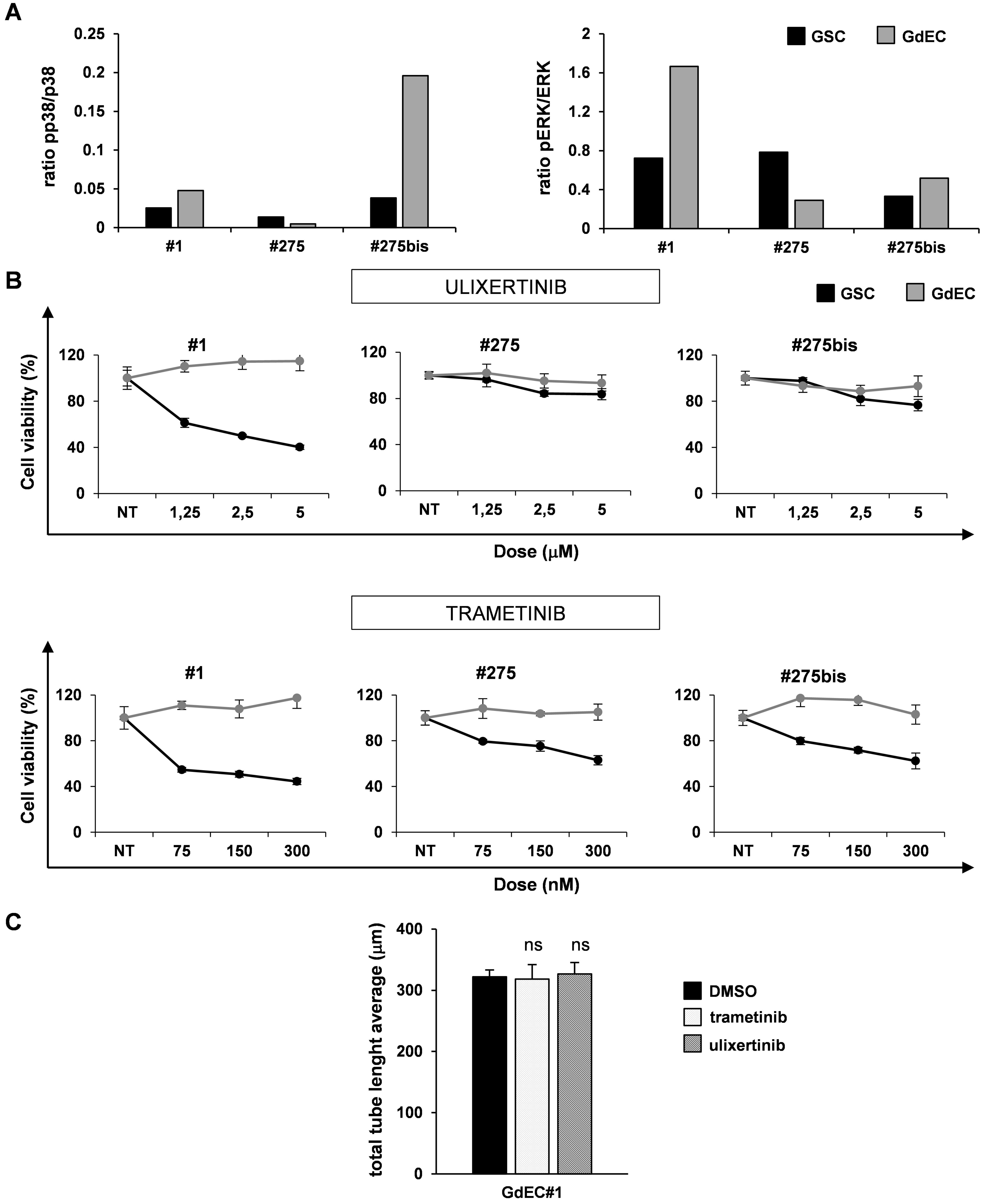

Supplementary Figure S4. **A** WES analysis of pp38/p38 and pERK/ERK ratio in three GSC lines (i.e. #1, #275, #275bis) and their GdEC counterpart. Signal intensity was normalized to the β-actin. **B.** Concentration-response assays of ulixertinib and trametinib were performed on three GSC lines and their GdEC counterpart. For each dose, mean values ± SD of residual cell viability normalized to control (NT) are reported and drug tested for 48 h. **C.** Quantification of total tube length average on tube-like structures formed by vehicle-treated GdEC#1 (DMSO) and GdEC#1 treated with trametinib (150nM) and ulixertinib (2,5µM) after 6h. ns non-significant (Student's *t* test).

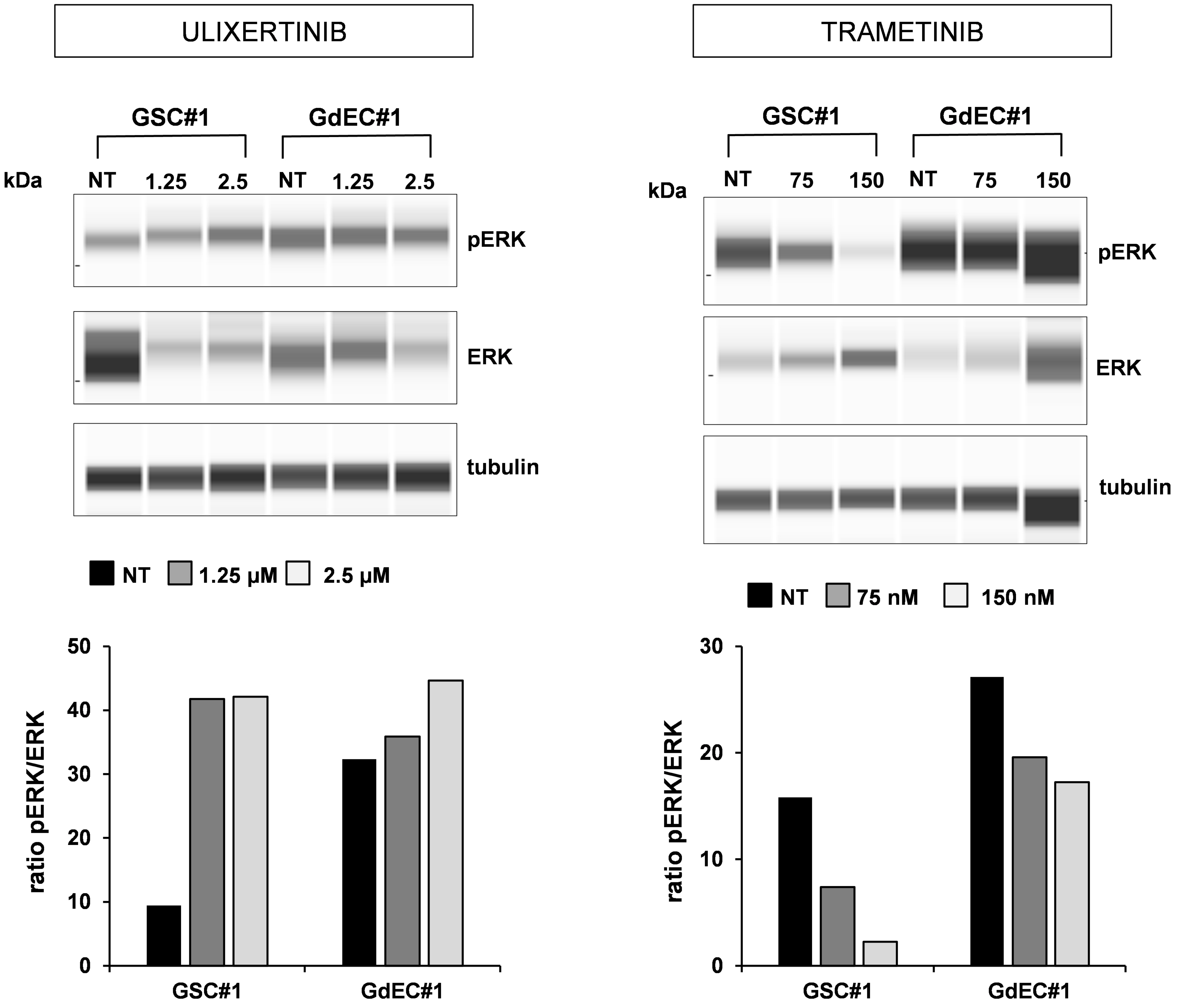

Supplementary Figure S5. WES analysis of pERK/ERK ratio in GSC#1 line and its GdEC counterpart treated with ulixertinib (1,25 and 2,5µM), (*left panel*) or trametinib (75 and 150nM), (*right panel*) after 48h. Signal intensity was normalized to the tubulin.

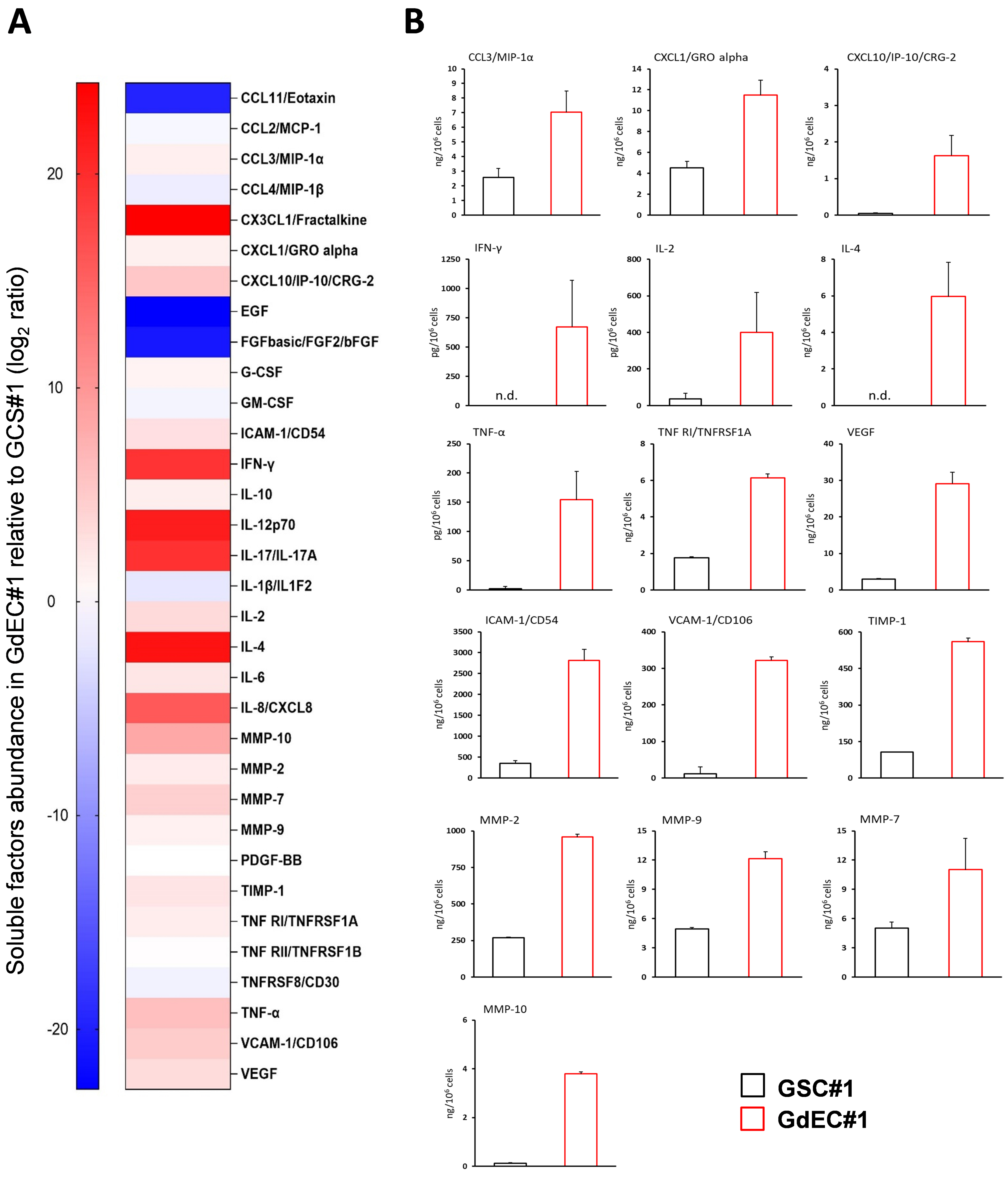

Supplementary Figure S6. A. Luminex analysis of cytokines, chemokines, metalloproteinases, and growth factors. Heat map of log2 ratios representing the comparison of the average secreted protein abundance between GSC#1 and GdEC#1. **B.** Histograms report only significantly different (p-value < 0.05) secreted molecules. Data are expressed as the mean ± SD of three different determinations.

A

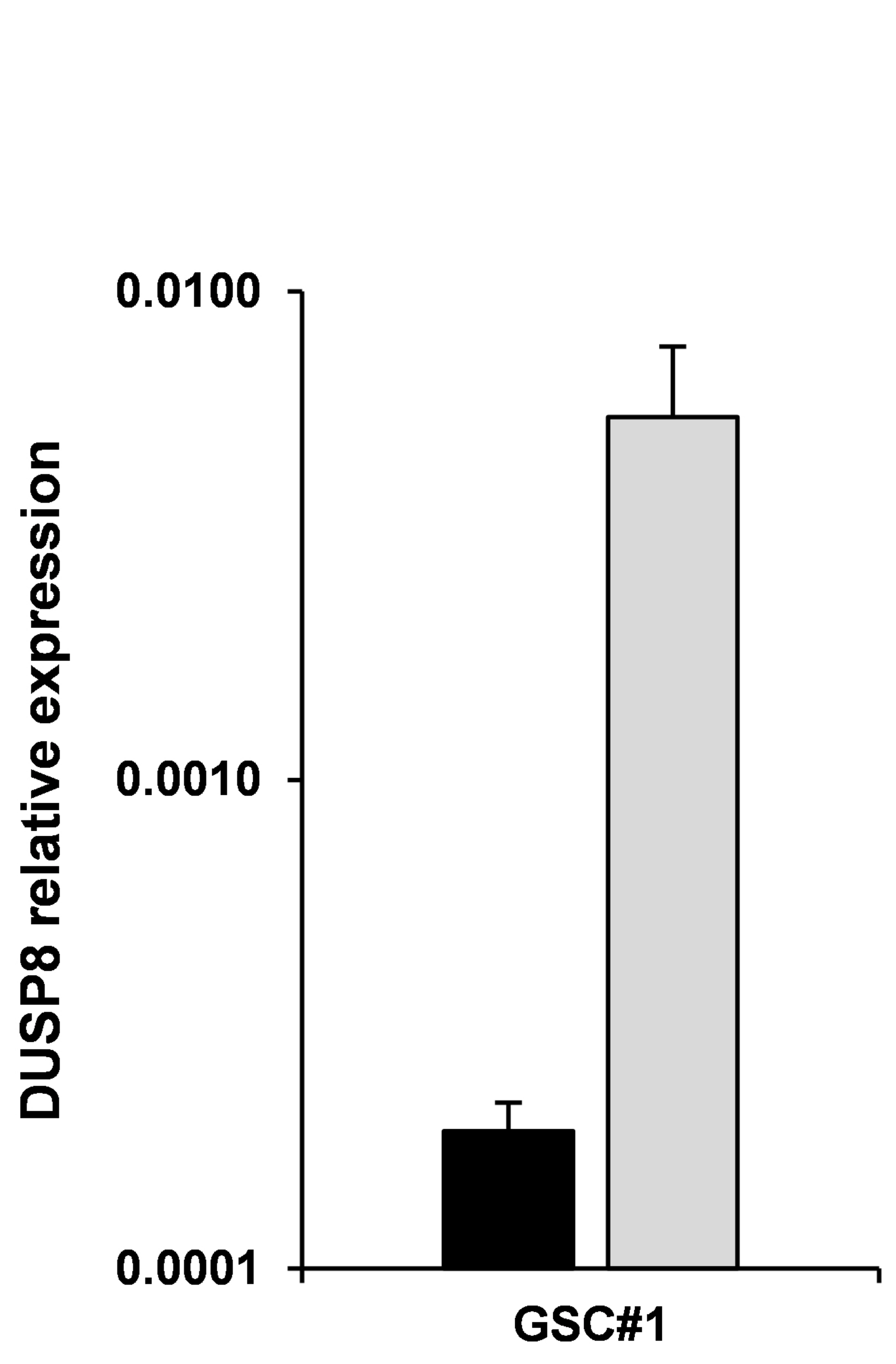

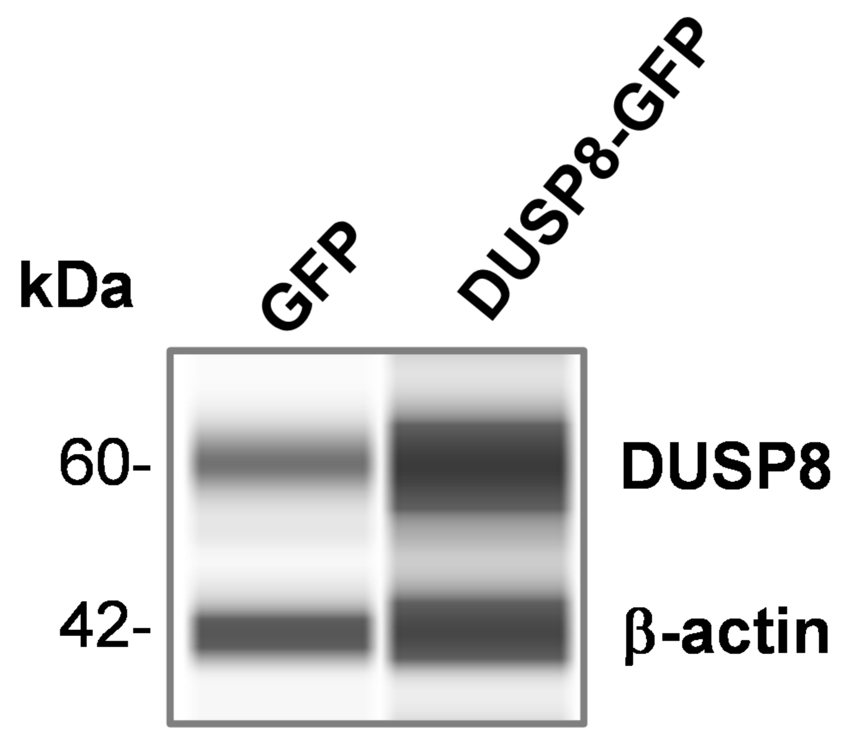

Sample	AUC	
	DUSP8	β-actin
GFP	360804,4	340226,5
DUSP8-GFP	897948,6	488885,3

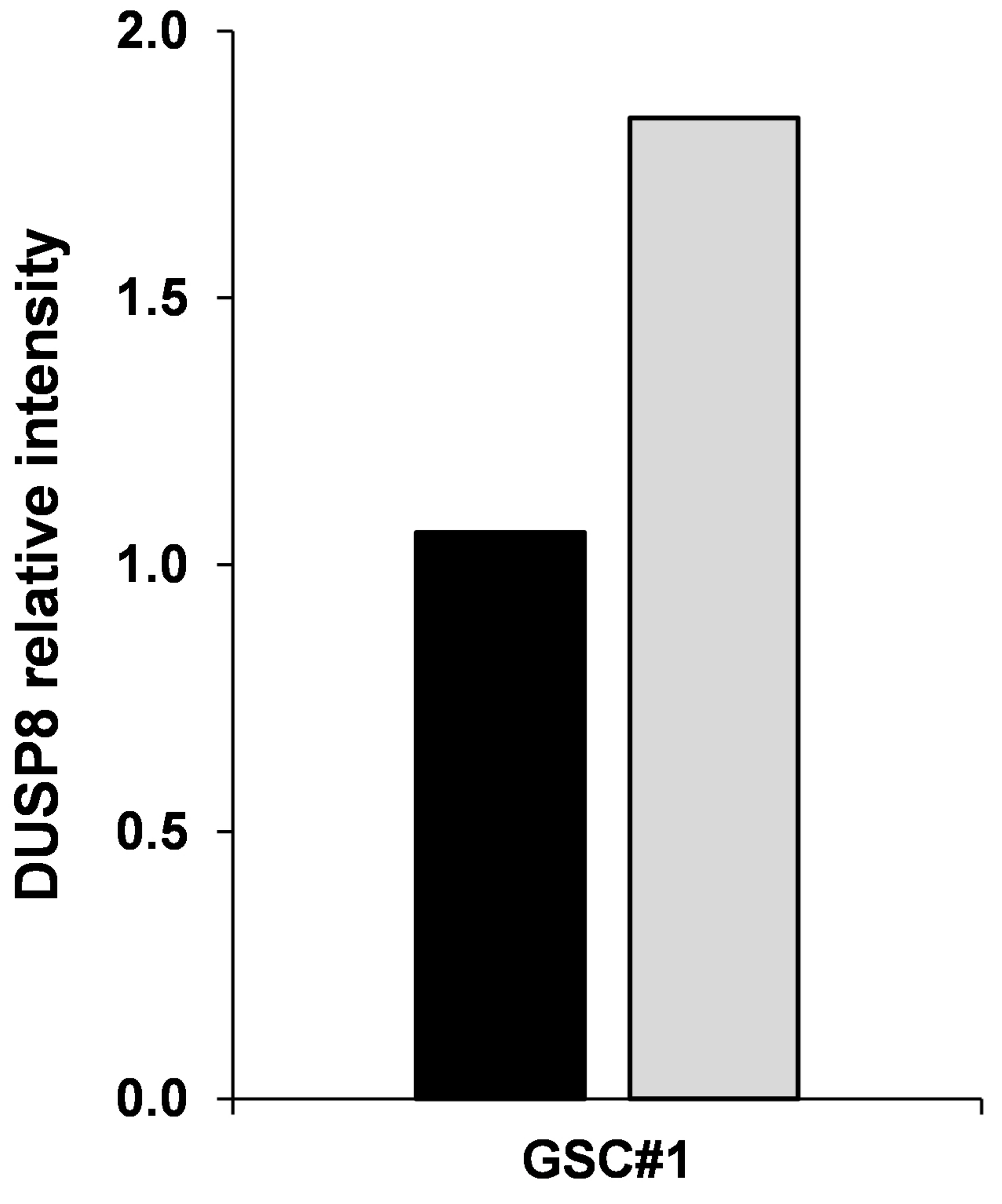

B

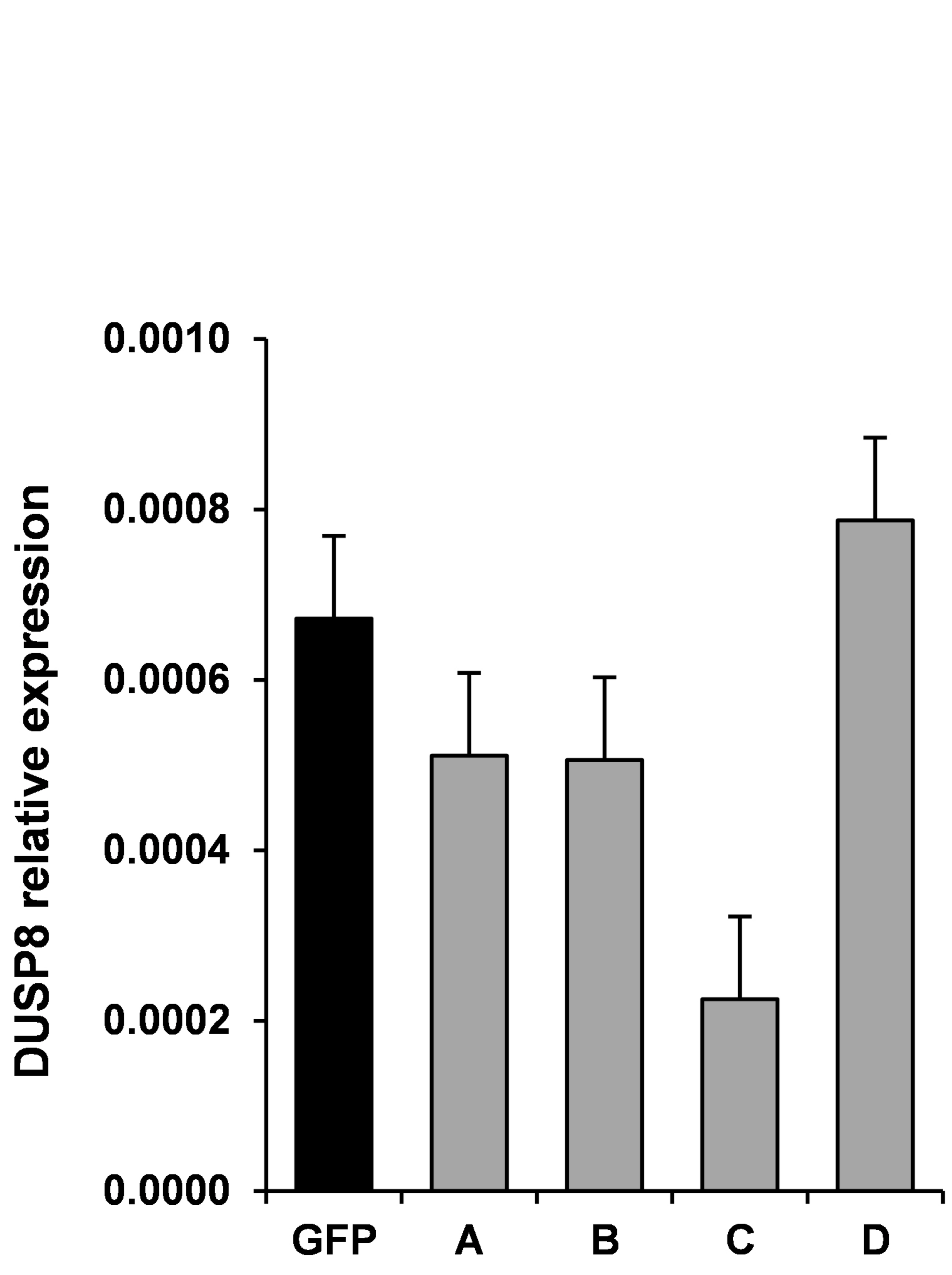

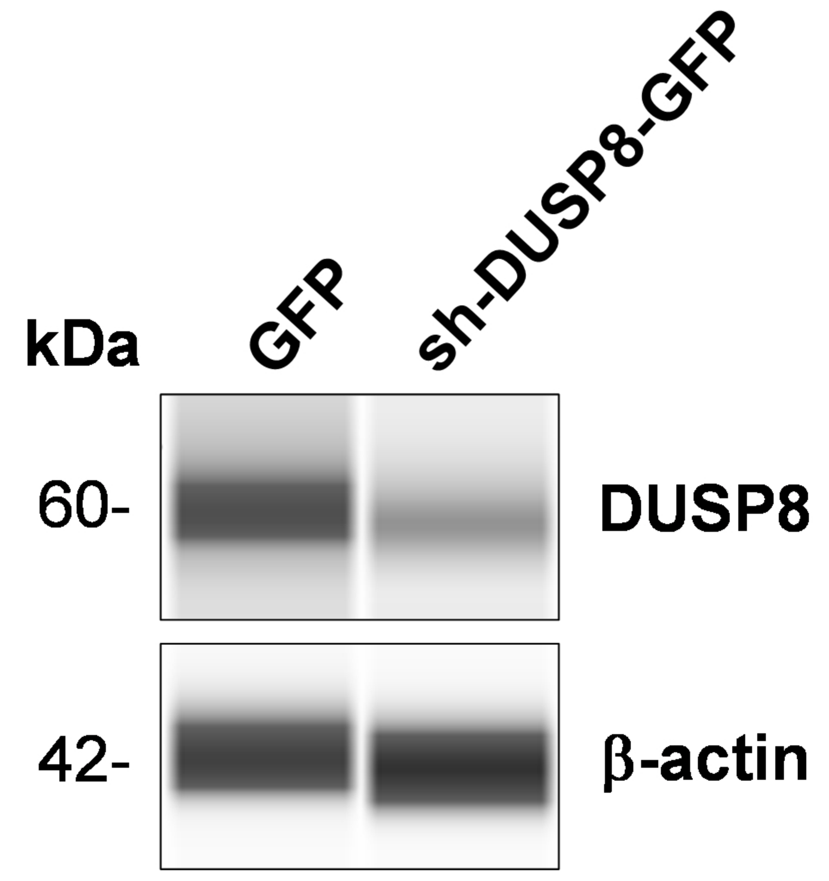

Sample	AUC	
	DUSP8	β-actin
GFP	94945,7	368804,4
sh-DUSP8-GFP	56564,4	410893

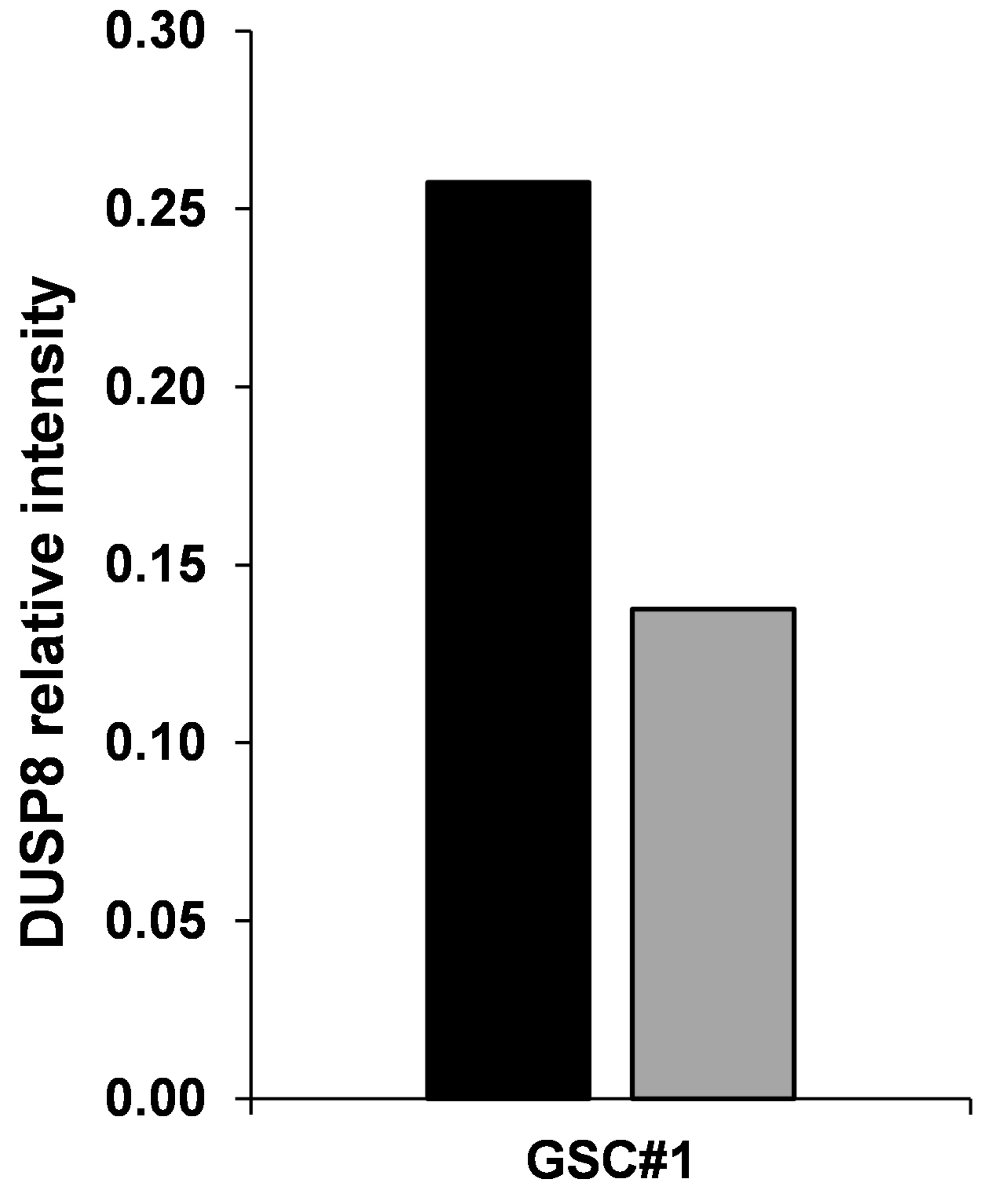

Supplementary Figure S7. **A.** RT-PCR analysis of DUSP8 expression in GSC#1 transduced with GFP or DUSP8-GFP vector (*left panel*). Samples were run in duplicate. Data were normalized to the GAPDH expression in the corresponding samples. WES analysis of DUSP8 expression in GSC#1 transduced with GFP or DUSP8-GFP vector (*center and right panel*). The protein expression was quantified using AUC measurements generated using Compass Software. The relative amount of each immunoreactive band was shown and signal intensity was normalized to the β-actin expression in the same sample. **B.** RT-PCR analysis (*left panel*) and WES analysis (*center and right panel*) of DUSP8 expression in GFP or sh-DUSP8-GFP GSC#1. For RT-PCR, samples were run in duplicate and data were normalized to the GAPDH expression in the corresponding samples. For WES, the relative amount of each immunoreactive band was shown, the protein expression was quantified using AUC measurements and signal intensity was normalized to the β-actin expression in the same sample.

A

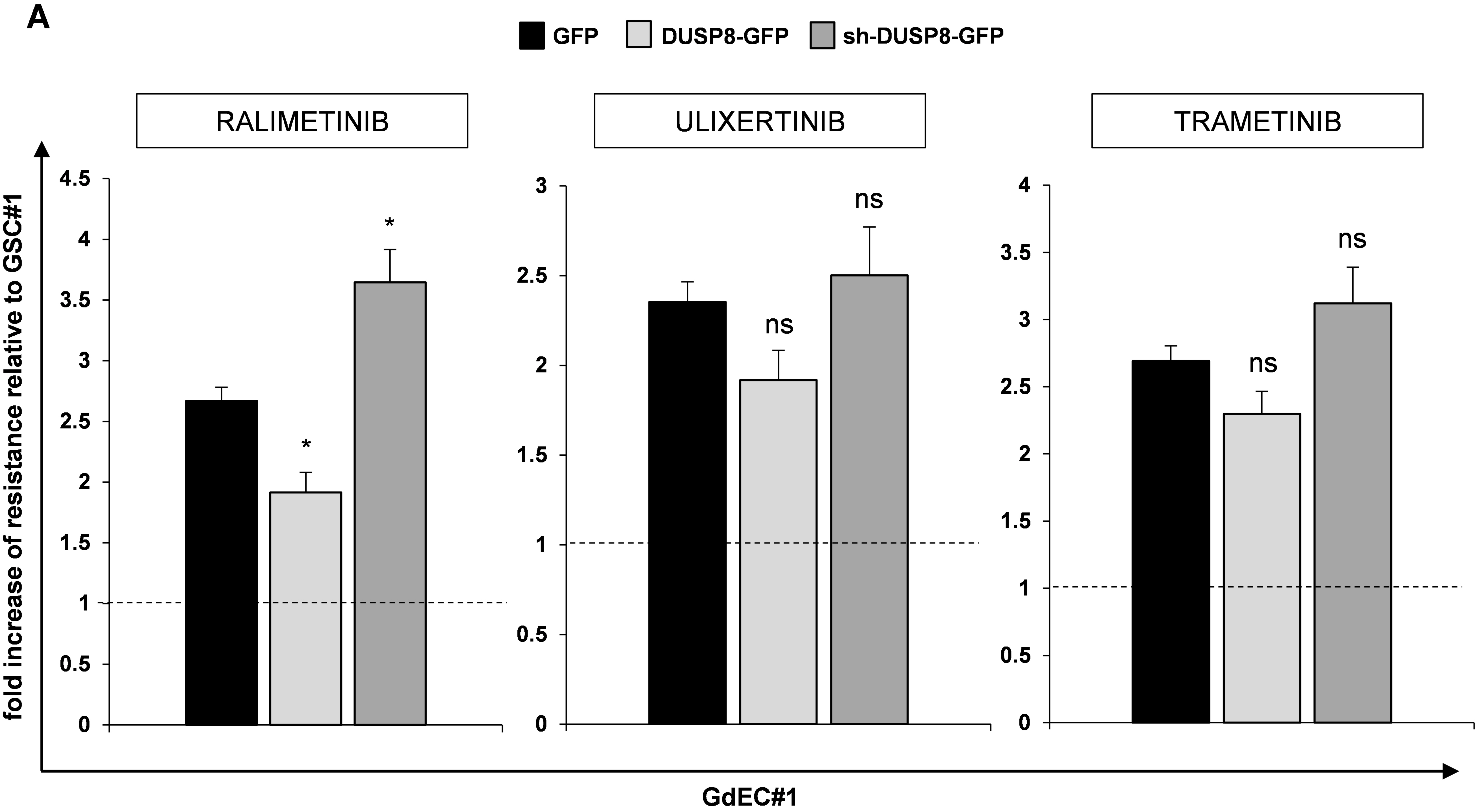

B

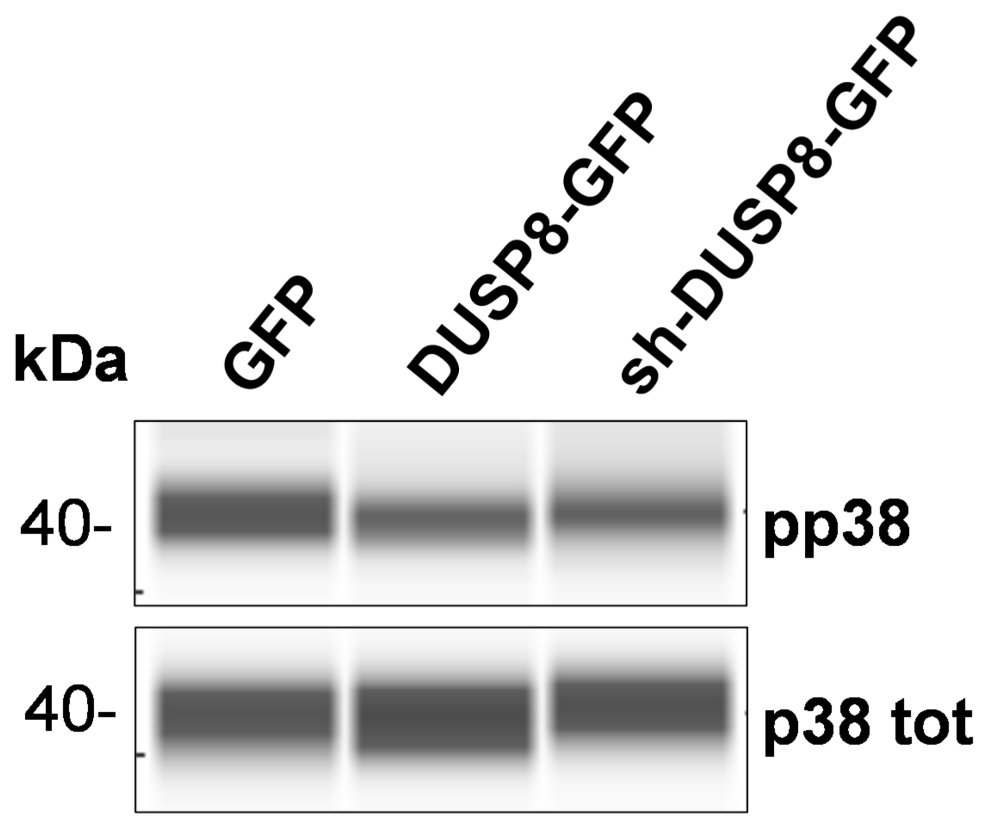

Sample	AUC	
	pp38	p38
GFP	26549	412629
DUSP8-GFP	24008	451136
sh-DUSP8-GFP	25666	402551

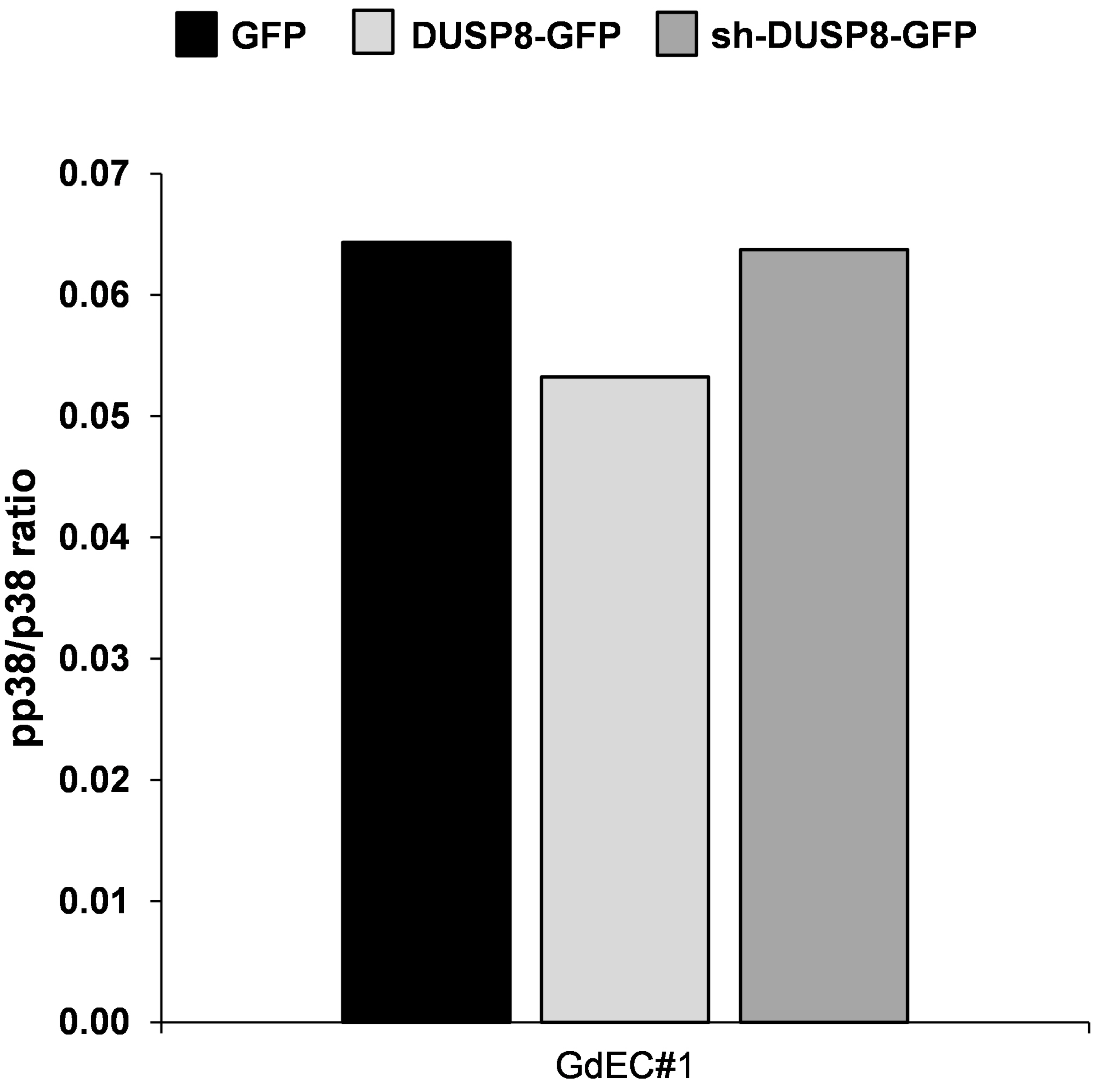

Supplementary Figure S8. A. Evaluation of cytotoxic effects of ralimetinib (40 μM), ulixertinib (5 μM) and trametinib (300 nM) treatment on transduced GdEC#1 after 48h (*n* = 2). Values are reported as fold increase of resistance relative to the transduced GSC#1 counterpart, used as reference (dashed line at value 1). *$p < 0.05$; ns= not significant vs GFP (Student's *t* test). **B.** WES analysis of pp38/p38 ratio in GdEC#1 transduced with GFP, DUSP8-GFP or sh-DUSP8-GFP vector. The protein expression was quantified using AUC (area under the curve) measurements generated using Compass Software. The relative amount of each immunoreactive band was shown and signal intensity was normalized to the β-tubulin expression in the same sample.

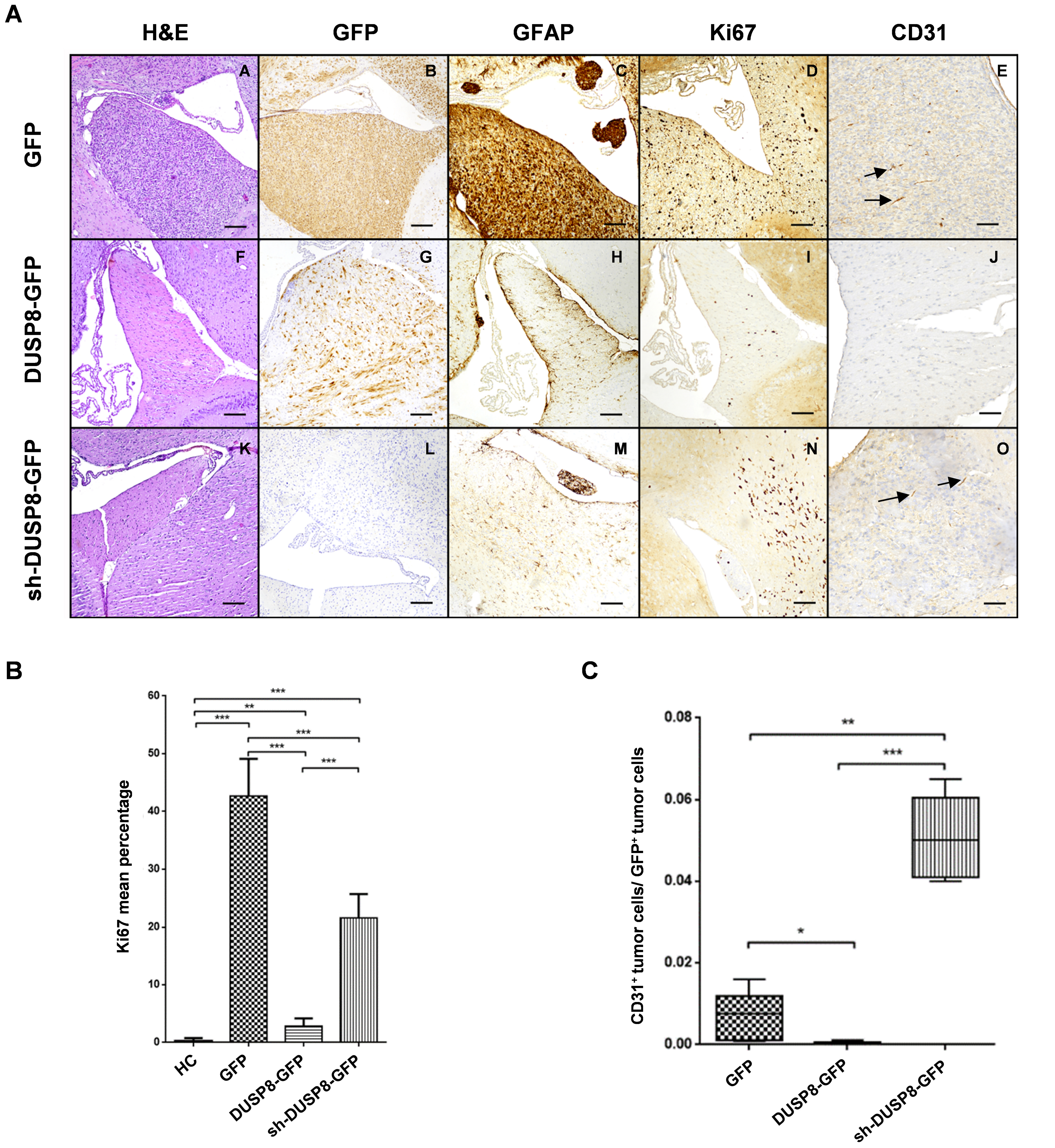

Supplementary Figure S9. A. Sagittal section of xenograft mouse brain (hippocampus, corpus callosum, ventriculum and thalamus zone) with GFP (panel A, H&E, 100x magnification), DUSP8-GFP (panel F, H&E, 100x magnification) and sh-DUSP8-GFP (panel K, H&E, 100x magnification), immunostained with anti-GFP antibody (panel B, G and L, respectively; 100x magnification), anti-human GFAP (panel C, H and M, respectively; 100x magnification), Ki67 (panel D, I and N, respectively; 100x magnification) and anti-human CD31 (panel E, J and O, respectively; 200x magnification). Arrows in panel E and O indicates the CD31 positive tumoral cells. **B.** Proliferative index (as Ki67 mean percentage) in sagittal section of xenograft mouse brain of HC mouse (healthy control; mean 0.09 ± SD 0.048), GFP mouse (mean 42.7 ± SD 6.4), DUSP8-GFP mouse (mean 2.8 ± SD 1.3) and sh-DUSP8-GFP mouse (mean 21.5 ± SD 4.1)**. C.** Ratio between CD31+ tumor cells and GFP+ tumor cells in sagittal section of xenograft mouse brain of GFP mouse (mean 0.067 ± SD 0.006), DUSP8-GFP mouse (mean 0.00026 ± SD 0.00042) and sh-DUSP8-GFP mouse (mean 0.051 ± SD 0.01).
